# Ecosystem-Wide Morphological Structure of Leaf-Litter Ant Communities along a Tropical Latitudinal Gradient

**DOI:** 10.1371/journal.pone.0093049

**Published:** 2014-03-26

**Authors:** Rogério R. Silva, Carlos Roberto F. Brandão

**Affiliations:** 1 Museu Paraense Emílio Goeldi, Coordenação de Ciências da Terra e Ecologia, Belém, PA, Brazil; 2 Museu de Zoologia da Universidade de São Paulo, São Paulo, SP, Brazil; University of California Riverside, United States of America

## Abstract

General principles that shape community structure can be described based on a functional trait approach grounded on predictive models; increased attention has been paid to factors accounting for the functional diversity of species assemblages and its association with species richness along environmental gradients. We analyze here the interaction between leaf-litter ant species richness, the local communities' morphological structure and fundamental niche within the context of a northeast-southeast latitudinal gradient in one of the world's most species-rich ecosystems, the Atlantic Forest, representing 2,700 km of tropical rainforest along almost 20^o^ of latitude in eastern Brazil. Our results are consistent with an ecosystem-wide pattern in communities' structure, with relatively high species turnover but functionally analogous leaf-litter ant communities' organization. Our results suggest directional shifts in the morphological space along the environmental gradient from overdispersed to aggregated (from North to South), suggesting that primary productivity and environmental heterogeneity (altitude, temperature and precipitation in the case) determine the distribution of traits and regulate the assembly rules, shaping local leaf-litter ant communities. Contrary to the expected and most common pattern along latitudinal gradients, the Atlantic Forest leaf litter ant communities show an inverse pattern in richness, that is, richer communities in higher than in lower latitudes. The morphological specialization of communities showed more morphologically distinct communities at low latitudes and species redundancy at high latitudes. We claim that an inverse latitudinal gradient in primary productivity and environmental heterogeneity across the Atlantic forest may affect morphological diversity and species richness, enhancing species coexistence mechanisms, and producing thus the observed patterns. We suggest that a functional framework based on flexible enough traits should be pursued to allow comparisons at local, regional and global levels.

## Introduction

Functional diversity is an important concept in community ecology because it encompasses information on functional traits, absent in measures of species diversity [1]; functional traits are morphological and physiological indicative qualities of the ecological strategies of species. The use of functional traits to quantify ecological similarities and differences among co-occurring species is offering new insights into community structure and assembly processes based on observational data [2]. The measure of functional diversity of a community through time has emerged as a key concept to explain ecosystem resilience to environmental change [3], to describe ecosystem processes [4] and the ecosystem services [5]. There are several studies on latitudinal gradients and large-scale environmental variables [6], [7], although there is no consensus on the determinants of species richness [6], [8]. The knowledge on the extent to which functional diversity is affected by latitudinal or environmental gradients is also lacking [9], [10]. The examination of functional diversity along extensive environmental gradients may provide complementary insights to those gained by the examination of species richness, and may help to identify general phenomena determining variation in biodiversity [11], [12]. The functional trait approach is particularly suited to address the role of summed species ecological strategies in shaping tropical forest biodiversity [2], [13] and enables appropriate description of communities because species-environment relationships are assumed to be mediated by functional traits [14], [15].

Invertebrate traits have been rarely used to study community response to habitat gradients [16], moreover the trait-based approach has been stressed as more quantitative and predictive to biodiversity studies than pure taxonomic comparisons [12], [17]. From a functional perspective, ants play fundamental roles in terrestrial ecosystems [18]. As consumers, they play a central position in the transfer of energy and cycling of nutrients [19]; because of their comparatively high density, ants may be considered keystone species in many ecosystems [20]. We focus on one of the most diverse ant faunas in the world (the leaf-litter fauna), along an extensive latitudinal gradient in one of the world's most species-rich ecosystems: the Brazilian Atlantic rainforest. We use data from 26 evenly spaced local ant communities to examine latitudinal patterns of species richness, morphological diversity and guild distribution. We quantified morphological structure of the leaf-litter ant fauna along the environmental gradient. If deterministic assembly rules influence community membership through trait-mediated species-species or species-environmental interactions, we should see their signals in the distribution of coexisting species phenotypes [21], [22], [23], [24]. Alternatively, absence of structured trait distribution in the ant communities suggest that stochastic processes may drive the shape of communities with respect to the analyzed ant traits. Finally, we examine the effect of selected climatic variables (temperature and precipitation), site characteristics (elevation and habitat area), and spatial data (latitude) on local ant species richness, morphology, and functional and beta diversities (taxonomic and functional). The strength of our study is that we were able to test the fit of models that describe community assembly processes along a latitudinal gradient using several approaches. Few large-scale tests of coexistence theories in tropical invertebrate communities have explicitly examined the ecological strategies of co-occurring species, in part because of the huge effort involved [25]. For instance, for the present study, we estimate that 260,000 ant specimens were collected, identified into genera and morphospecies and 78,000 species representatives pin-mounted for documentation purposes.

## Materials and Methods

### Ethics Statement

All necessary permits required for the described study were obtained, complying with all relevant regulations. All research was conducted with the approval by the Brazilian Institute of Environment and Renewable Natural Resources (IBAMA, Permit Number 048/2002, Proc. 02001.000796/01-52).

### Ant sampling

**Figure 1:**
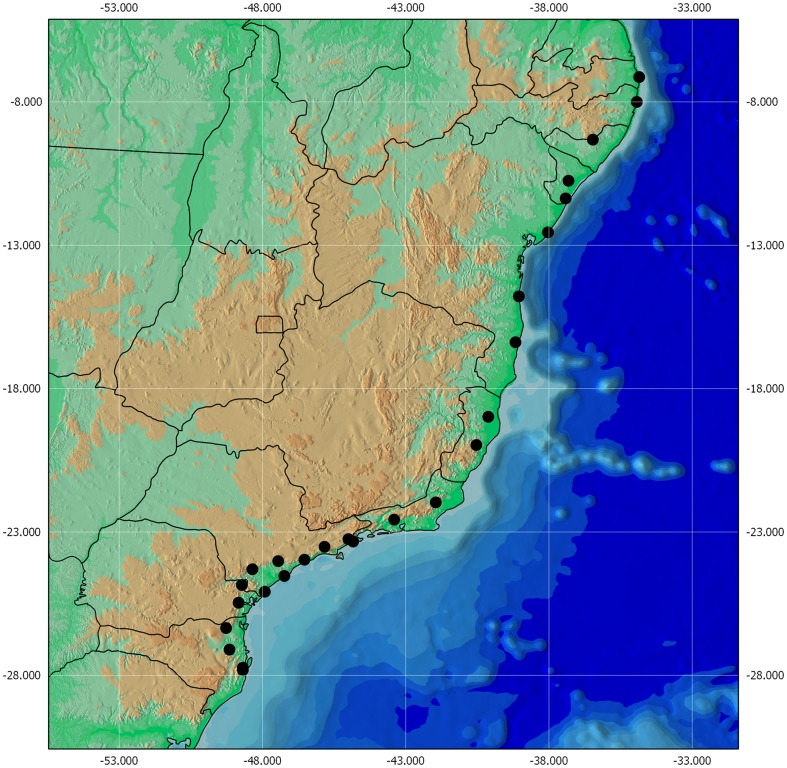
Distribution of sites along a latitudinal gradient of Atlantic Forest, Brazil.

We surveyed 26 Atlantic rain forest localities (Fig. 1 and Table S1) as regularly spaced as possible. The Atlantic rain forest covers mostly low to medium elevation areas (≤1000 m above sea level) on the Eastern Brazilian coastline, with a warm and wet climate without a true dry season [26]. The biome covers a wide range of latitudes (from 6 to 30 degrees S), altitudes (from zero to 2,900 m) and climates (from tropical to subtropical). Our sites are within representative conservation units of the Atlantic Forest system, between 19 and 1082 m a.s.l., in mature forests classified as lowland (<300 m), submontane (300–700 m) and lower montane rain forests (700–1100 m) [26]. The Atlantic Forest shows relatively higher altitudes only south of mid Espírito Santo, so the remnants we choose at high altitudes are all in the southern portion of the biome (eight sites), were we sampled also remnants in lower altitudes (0–300 m).

We carried out quantitative surveys in the years 2000 and 2002 summers (December-March), when the ant activity is believed to be relatively high. Along each site 50 1-m^2^ leaf litter samples were collected and mini-Winkler apparatuses were used to extract the ant fauna. At each site, a transect of 1,200 m was selected for the ant survey, starting at least 500 m from the forest edge. At each transect we marked 25 points set at 50 m intervals; at each point, two 1-m^2^ leaf litter samples were collected, one 25 m to the left and one 25 m to the right of the point, covering a projected sampled area of 12.5 hectares. In total, fifty leaf litter samples were collected at each site, vigorously sifted through a sieve of 1 cm grid size and transferred to mini-Winkler extractors, where the sifted leaf litter was held in a mesh sack and suspended in a larger cotton enclosure for 48 hours. The ants dropped out of the mesh sack to be collected live in plastic cups containing a humid sponge. The ants were then transferred to white trays and then to vials with 70% ethanol. Sampling was carried out between 9:00 a.m. and 4:00 p.m. The ants of all samples were sorted out and up to three workers of each morphospecies in each sample were mounted. Whenever possible, specimens were identified to species by comparing them to taxonomic descriptions and material deposited in the Museum of Zoology of the São Paulo University Formicidae collection, where voucher species were deposited. Specimens not identified to species were given a code for each distinct morphospecies.

### Ant morphological data

We measured morphological variables of 1,944 individuals from 530 species, extracted from leaf litter samples collected in the Atlantic Forest localities. Sample sizes for morphological studies ranged from one to ten individuals per species (average  = 5/species), depending on availability and within species size ranges. There were 128 species represented by a single individual, 63 species by two, 44 species by three, 33 species by four, 215 species by five and 47 species by six or more measured individuals.

We selected nine ant morphological characters relevant to indicate, isolated or in groups, habitat preference and resource use [27], [28]: (1) mesosoma length, as a surrogate of total length, a key trait to many life histories of ants [29], [30]; (2) mandible width, related to resource size; (3) compound eye length, important in food search; (4) distance of compound eye to mandible insertion, imposing different performance for visual predatory species; eye position has been documented as comparatively closer to mandibles in predatory species (e.g. trap-jaw mandible species) [31]; (5) interocular distance (in general predatory ant species have compound eyes set more distant on the head capsule than in other species); (6) clypeus length which is linked to the trophic level of species (genera that rely strongly on liquid food have in general highly modified clypeus); (7) hind femur length, assuming that leg size may be related to species distribution inside the leaf-litter [29], [30]; (8) petiole length and (9) petiole height, correlated to predaceous species behavior and performance [28]. The same person (Rogerio R. Silva) performed all measurements. The morphological data set is available in the Supporting Information (Table S2).

### Environmental data

We obtained environmental data from Worldclim (http://www.worldclim.org) at 30 s resolution. At each locality, five variables were chosen to represent climatic data: (1) annual mean temperature, (2) minimum temperature (the average minimum temperature of the coldest month), (3) maximum temperature (the average maximum temperature of the warmest month), (4) temperature annual range (defined as the difference between the maximum temperature of the warmest and coolest months); and (5) annual precipitation. We used (6) altitude (elevation above sea level) and (7) habitat area (hectares) as site characteristics (Table S2). Elevational data was derived from the three arc-second Shuttle Radar Topography Mission (SRTM) Digital Elevation Model [32]. Elevation range has been used as a measure of environmental heterogeneity in the analysis of the ecological communities [33]. In addition, we considered (8) annual actual evapotranspiration (AET) and (9) annual potential evapotranspiration (PET) as proxies for primary productivity and ambient energy input, respectively [7]. AET and PET were derived from the Global High Resolution Soil-Water Balance with 30 arc seconds resolution [34]. We used climatic and environmental variables that are strongly correlated with species richness in ant studies [35] and are important variables of species energy theory [36].

### Species richness and functional diversity analysis

Species richness was defined as the number of species recorded in each locality. We used the morphological traits to estimate morphological diversity. Trait variation or multivariate trait differences within a community is generically referred to as ‘functional diversity’ [17]. For each species, mean trait values were computed from individual measures and used to calculate functional diversity. We used four multidimensional functional diversity indices, each exploring a different aspect of the functional diversity: functional richness (FRic), functional evenness (FEve), functional divergence (FDiv), and functional specialization (FEsp) [15]. The multidimensional FRic index measures the amount of functional space filled by a community, calculated as the convex hull volume. The FEve index measures the regularity of the abundances distribution in a multidimensional trait space. The FDiv index expresses the degree in which the abundance distribution maximizes the spread of functional characters within the trait space, measuring the distance of the most abundant species to the center of gravity in the functional space. Further, we used the functional diversity index of Petchey and Gaston [37], referred to as FD_PG_, which measures the total branch length of a community functional dendrogram and represents the functional complementarity among species (the larger the functional differences between species, the larger will be FD). We used the *FD_Ind* function [15] <http://www.ecolag.univ-montp2.fr/software>) for computing the components of functional diversity. FD_PG_ was computed using the *treedive* function in the vegan package [38].

### Predictors of taxonomic and functional diversities

First, we assessed the relationship between morphological or taxonomic diversity and latitude with generalized linear models (GLMs). Model checking showed data heterogeneity (more variation at greater latitudes) and non-linearity related to altitude; so we used GAM models with Gaussian distribution, latitude and altitude as predictors and a fixed variance structure in the model, allowing for larger residual spread if altitude increases.

Then, we used a multiple-predictors model to test the effects of climatic data (annual temperature range and mean annual precipitation), productivity (AET), habitat area and altitude on taxonomic and morphological diversity. The relationship between the morphological diversity indices and the predictors was assessed with regression models assuming a Gaussian error distribution. We used GLM Poisson with log-link function to model the relationship between species richness, rarefied species richness, abundance and the predictors. We used stepwise selection by AIC in the MASS package in R [39] to determine the best model in the multiple-regression analyses. When the deviance analysis suggested overdispersion in the multiple-predictors model, we used negative binomial GLM model because AIC values are undefined in quasi-Poisson GLM.

Because comparatively high altitude areas occur only in the southeastern of the Atlantic Forest, we refit the models excluding high altitude sites (eight sites higher than 400 m) to evaluate if the interpretation of latitudinal effects change when analyzing only lowland sites. When the deviance analysis suggested overdispersion in the single-predictor model, we adjusted standard errors using quasi-Poisson GLM.

### Functional and taxonomic beta diversity analysis

We used three metrics of functional beta diversity, each highlighting different aspects of functional turnover. The Functional Sorensen's Index (F_sor_) weighted presence-absence and is defined as: F_sor_  =  BL _k1 k2_/(BL_k1_ + BL_k2_)∧2, where BL_k1 k2_ is the total dendrogram branch length common to all species in communities *k1* and *k2*, and BL_k1_ and BL_k2_ are the total dendrogram branch lengths common to the species within communities *k1* and *k2*, respectively. The second and third used measures were the mean pairwise distance (F_MPD_) between all species and the mean nearest-neighbor distance (F_MNTD_) separating each species from its closest relative. We used the *comdist* and *comdistnt* functions in the picante package [40] to compute the mean morphological distance or mean nearest taxon distance between pairs of species drawn from two distinct communities.

For consistence with the F_sor_, we employed the Sorensen's Index (S_sor_) to calculate species turnover, which reflects the compositional dissimilarity between pairs of sites as the likelihood that a species occurs in these two sites: S_sor_  =  (b+c)/2a+b+c [41].

We used distance-decay relationships to assess rates of decline in taxonomic and functional similarity as a function of geographic and environmental distances [42]. We assessed the correlation of taxonomic and functional similarity of communities with geographic distance and environmental distance using partial Mantel tests [43].

We combined the climatic and environmental data (nine variables in total) in a principal component analysis to define the environmental gradient. The first principal component, used to examine the combined environmental turnover along the gradient, accounted for 60% of the variance in the environmental space and loaded the variables approximately equally (loadings: altitude = 0.407, annual mean temperature  = −0.453, maximum temperature  = −0.426, minimum temperature  = −0.445, temperature annual range  = 0.373, PET  = −0.317), except precipitation (loading  = −0.074) and AET (loading  = 0.050). The environmental distance between sites was calculated as the absolute difference between the values of the PC-1 at these sites. The range of temperature for the latitudinal gradient was 9.6 to 18.7 degrees Celsius, while it was 9.6 to 18.4 degrees Celsius to the lowland areas.

In addition to Mantel tests, we used distance-based redundancy analysis (dbRDA) coupled with permutation-based model selection to find the variables that most strongly relate to patterns of turnover along the latitudinal gradient [44]. Six variables were used to generate the “full” model in dbRDA (functional or taxonomic distance between sites ∼ Habitat Area + Altitude + Precipitation + Temperature Annual Range + PET + AET). The *capscale* function was used to carry out db-RDA and model selection was based on *ordiR2step* function in vegan package [38].

### Morphological structure analysis

We used two morphological structure metrics: the mean pairwise distance (MPD) and the mean nearest-neighbor distance (MNTD) among all species in an ant community to describe community assembly. The input data in the form of a distance matrix was built based on the Euclidean distance between species using the nine untransformed morphological ant traits.

A null distribution was generated by randomizing the names of the species across the tips of a trait dendrogram to assess the significance of observed MPD and MNTD. We used standardized effect sizes (SES) to express morphological structure: positive or negative values of these indices indicate morphological clustering or overdispersion. SES values greater than 1.96 or below than 1.96 indicate higher than expected s or lower than expected morphological dissimilarity between species, respectively. Values in between indicate no morphological structure.

Deviations from the null expectation can be used as evidence for action of a number of ecological processes determining the assembly of the local ant communities: (i) a regular or even dispersion of morphology is the predicted outcome of the classic concepts of competitive exclusion or niche partitioning [21]; (ii) according to the “habitat filtering” hypothesis, co-occurring species that share a similar set of adaptations to the environment share also the same set of traits related to resource use [13]; (iii) convergent evolution can lead to similar trait values within communities; (iv) competition can also produce trait convergence by excluding dissimilar species [45]; (v) finally, non structured morphology may indicate neutral processes determining community assembly (random patterns in the functional identities of co-occurring leaf-litter ant species resulting from regionally or locally important stochastic processes).

We did not translate the predictions of existing ant diversity hypothesis into a set of estimates of the trait-based tests applied in this study. We were interested in the analysis of spatial variation in the morphological structure of leaf-litter ant communities, and not to infer the processes underlying filters, but rather to quantify their relative strength and direction. To reach this, we calculated MPD and MNTD indices of “random communities”, each defining a different species pool to the null communities: (1) unconstrained Atlantic Forest species pool (randomizing site composition across all sites); (2) constrained within-region species pools (randomizing site composition across Atlantic Forest regions); (3) unconstrained within-guilds (randomizing within-guild composition across all sites); (4) constrained within-guilds (randomizing within-guilds composition across Atlantic Forest regions). In the unconstrained analysis, trait dissimilarity within communities (or guilds) is compared to the corresponding trait dissimilarity expected for the Atlantic Forest list; in the constrained analysis, the expected trait dissimilarity is calculated for Atlantic Forest species pools (different regions of the biome or subsets in the data set as determined by an UPGMA clustering based on Bray-Curtis distance index) that include the set of potential species for a site delimited likely by environmental and dispersal limitation filters. Visual examination of the cluster indicates that sites can be grouped into four groups: (1) north localities (8 sites, 232 species), (2) intermediate latitude areas (4 sites, 236 species) (3) low southeastern-south localities (8 sites, 236 species), and (4) high southeastern-south Atlantic Forest areas (6 sites, 263 species).

The species pools' sizes were defined as the number of species in each group determined by the cluster analysis. In all randomizations we kept the number of species in a community (or sample) the same. Although our definition of species pool is simple (see applied definitions in ref. [46]), the pools determined for each Atlantic Forest region likely include all species that could disperse to a given location at the Atlantic Forest within the same region and also agree with most definitions of sub-biomes in the Atlantic Forest [47], [48]. Our analyses were designed to quantify morphological structure across the latitudinal gradient and under different scales of species coexistence. It is important to note that each species pool was built to investigate a different question, so the consistency or inconsistency in results among scales provides insights on the related ecological processes. For example, we predict that the processes generating even species trait spacing will be more important at small spatial scales and within guilds, where neighbors interact and compete for resources, as well as at scales in which negative density-dependence is likely to have significant impacts on species co-occurrences.

We use picante package [40] in all analyses. We then fit a GAM to evaluate the relationship between latitude, MPD and MNTD values by using the mgcv package in R [49], with fixed effects (latitude) modeled as smooth functions (thin plate splines).

## Results

### Species richness and functional diversity across the latitudinal gradient

**Figure 2:**
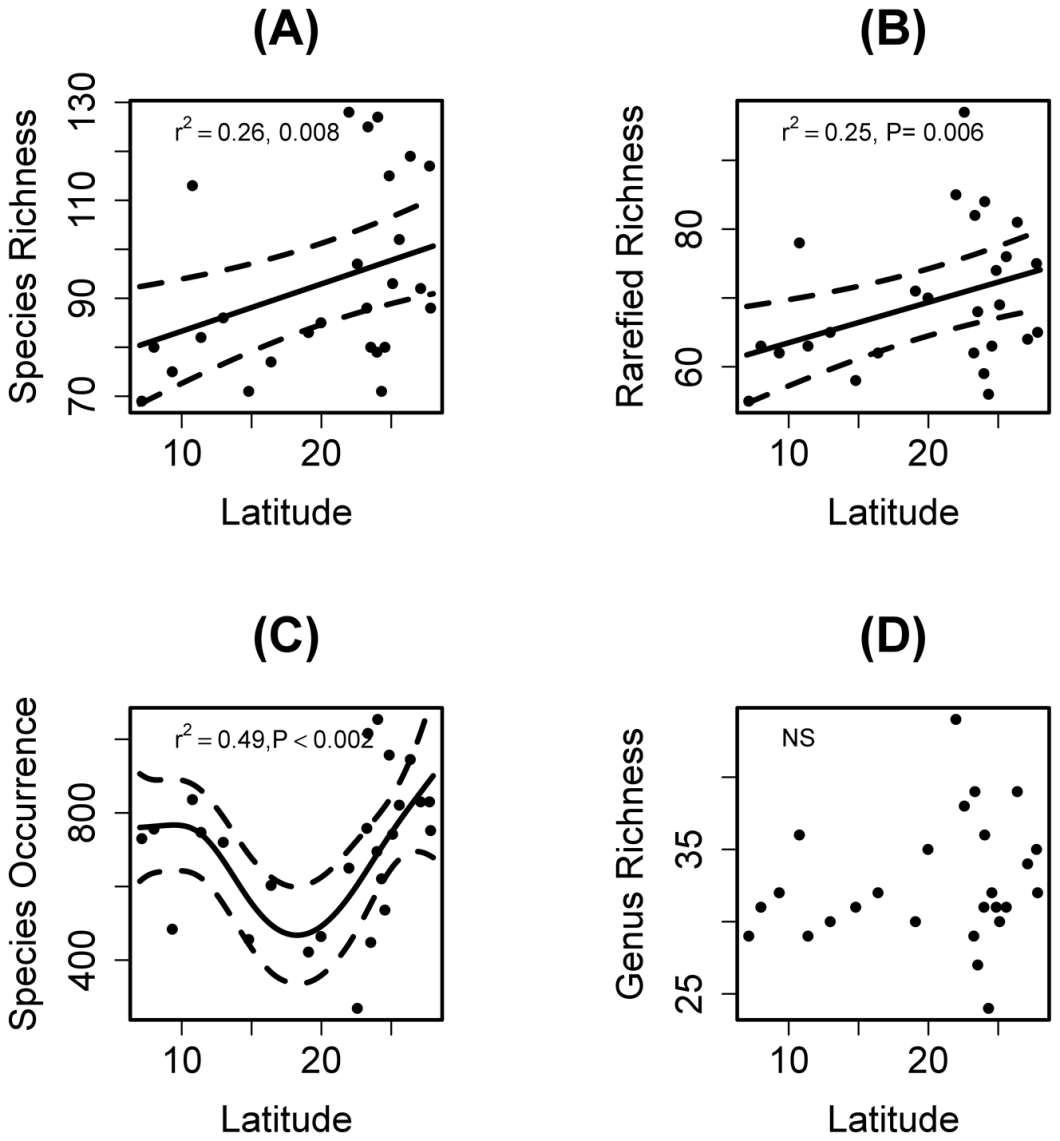
Relationship between latitude along the Brazilian Atlantic Forest and (A) observed leaf-litter ant species richness, (B) rarefied species richness, (C) number of ant genera, (D) and number of species occurrences. The dotted lines represent 95% point-wise confidence intervals and the solid line is the estimator in the GAM for the smoothing function *f(Latitude)*.

**Figure 3:**
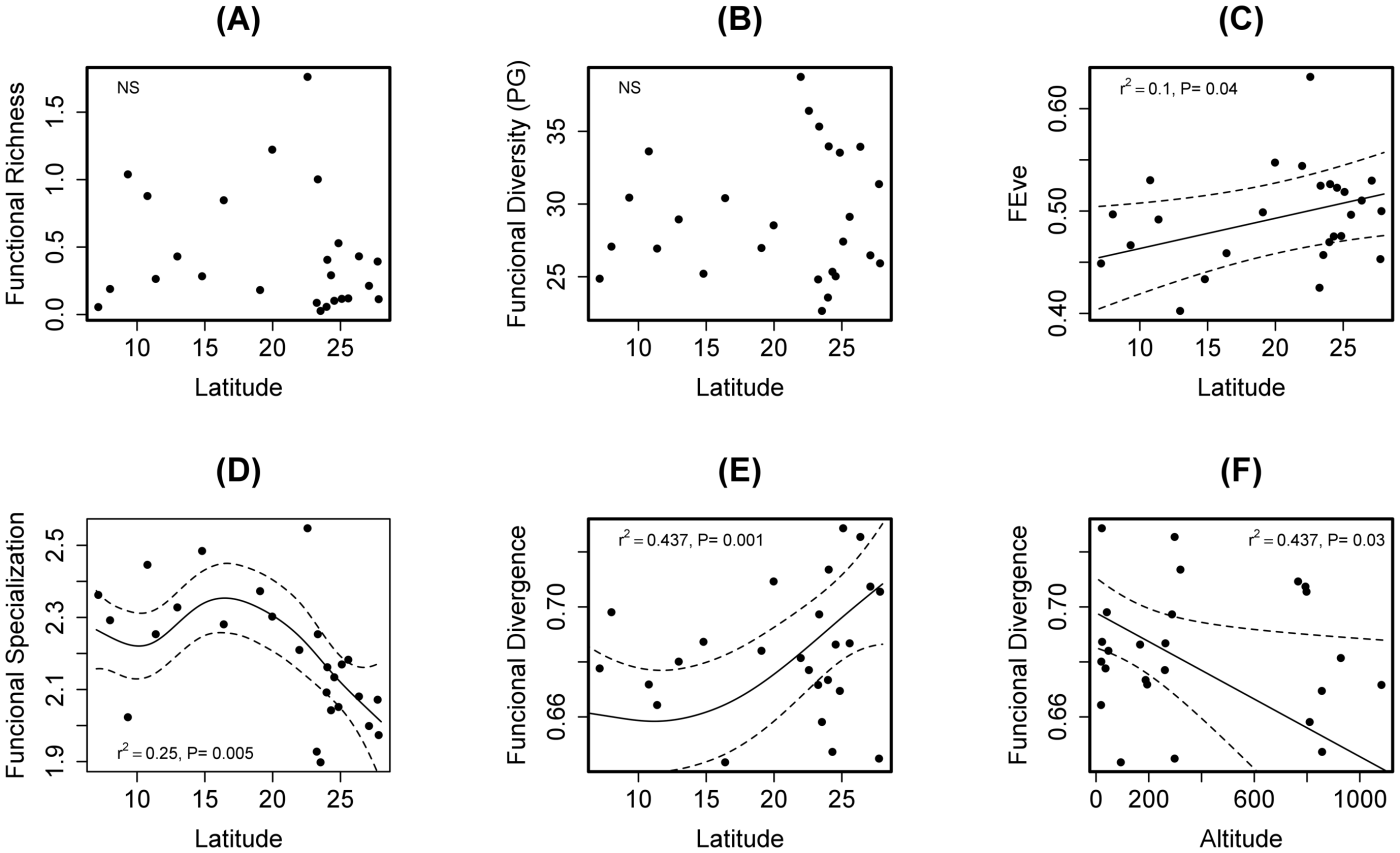
Relationship between latitude and components of leaf-litter ant functional diversity along the Brazilian Atlantic Forest. Graph (A) functional richness (FRic), (B) functional diversity of Patchey and Gaston (F_PG_), (C) functional evenness (FEve), (D) functional specialization (FEsp), and (E–F) functional divergence (FDiv). The dotted lines represent 95% point-wise confidence intervals and the solid line is the estimator in the GAM for the smoothing function *f(Latitude)* or *f(Altitude)*.

Altogether we registered 18,142 ant records in 1,300 1-m^2^ samples of leaf-litter in 26 areas covered by Atlantic Forest. Overall, we identified 530 ant species in the leaf-litter. Observed species richness showed a weak inverse latitudinal pattern (Latitude: F_1,23_ = 8.330, P = 0.008; Adjusted R-square  = 0.258; Fig. 2A), even after correcting for number of species occurrences (rarefied species occurrences, Latitude F_1,23_ = 8.960, P = 0.006; Adjusted R-square  = 0.248; Fig. 2B). The number of species occurrences of the leaf-litter ant fauna showed a non-linear relationship with latitude (F_3.7, 21_ = 6.468, P = 0.001, Adjusted R-square = 0.49; Fig. 2C); number of ant genera did not show significant relationship with latitude (Fig. 2D, Table S3-A). The analysis considering lowland Atlantic Forest sites show the same results suggesting a latitudinal effect on taxonomic richness, although the deviance explained by generalized linear models increased when altitude was controlled (Table S3-B). Functional diversity components (richness, divergence, evenness, specialization) showed different responses to the Atlantic Forest latitudinal gradient. Functional richness measures (how much of the niche space is occupied by the species in the communities) were not related to latitude (FRic: F_1,23_ = 0.333, P = 0.570; FD_PG_: F_1,23_ = 0.489, P = 0.491; Fig. 3A–B). There was also a weak relationship between functional evenness (how regularly species abundances are distributed in the functional space) and latitude (FEve: F_1,23_ = 4.644, P = 0.041; Adjusted R-square = 0.108; Fig. 3C). Functional specialization of the communities showed a non-linear pattern with latitude, decreasing to mid and high latitudes (Lat F_3.7,20_ = 7.063, P = 0.001; Adjusted R-square  = 0.59; Fig. 3D); functional divergence (how far high species abundances are away from the center of the functional space) showed a slightly non-linear relationship with latitude and negative linear relationship with altitude (Latitude F_1.9,21_ = 9.760, P = 0.001; Altitude F_1,24_ = 4.941, P = 0.037, Adjusted R-square = 0.437) (Fig. 3E–F). Models considering lowland sites did not show, however, relationship between latitude and functional characteristics of the communities, suggesting that the non-linear relationships may be determined by an altitudinal effect.

### Predictors of taxonomic and functional diversities

Stepwise selection of the multiple-predictors models identified temperature as the best variable explaining leaf-litter ant species richness along the Atlantic Forest. The best model for rarefied species richness retained temperature, altitude and precipitation as predictors (Table S4-A). There was no relationship between abundance and climatic or environmental predictors. The best models for taxonomic diversity accounted for 20% and 26% of the variation in observed and rarefied species richness respectively. The best models for functional diversity indices retained consistently temperature, precipitation, altitude and productivity as best predictors (Table S4-A), accounting, however, for only 12% (FD_PG_), 5% (FRic), 9% (FEve), and 16% (FDiv) of the variation in functional diversity. Up to 53% of the variation in the functional specialization of the communities was explained by a combination of temperature, altitude, productivity and precipitation. Habitat area was consistently excluded in the best models suggesting no effect of habitat area in the scale of our study on functional or taxonomic diversities (Table S4-A). Overall, excluding high altitude areas from analysis did not change interpretation of models (Table S4-B); however, similar to the single-predictor models there were no relationship between functional richness indexes and environmental variables.

### Functional and taxonomic beta diversities

**Figure 4:**
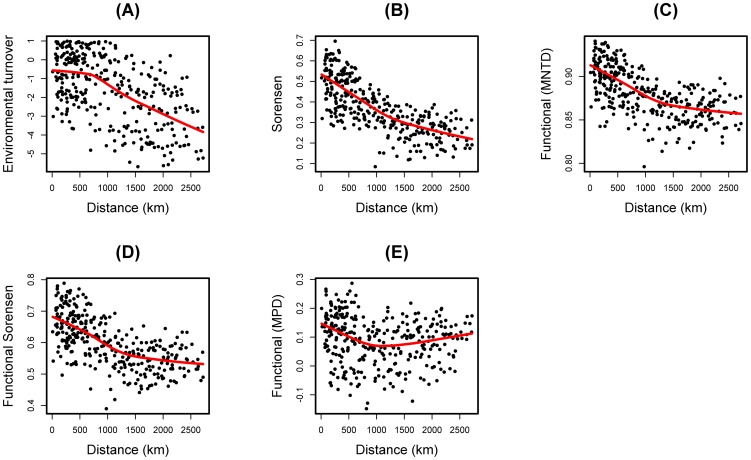
Relationship between environmental distance along the Brazilian Atlantic Forest and (A) geographic distance, (B) taxonomic beta diversity (Sorensen's Index); (C) functional beta diversity (MNTD), (D) functional Sorensen's beta diversity, (E) and functional beta diversity (MPD).

A strong correlation between environmental and geographic distance was revealed by the data (Mantel r = 0.56, P = 0.001), with adjacent sites being most similar between each other than to any other site in terms of environmental conditions (Fig. 4A). We therefore used partial Mantel tests to assess the influence of environmental distance (measured by selected environmental variables) on community taxonomic (or functional) similarity, while holding the geographical distance constant. The beta taxonomic diversity and two functional beta diversity indices (MNTD and functional Sorensen's) showed a general decay in similarity with geographical or environmental distance along the Atlantic Forest (Fig. 4B–D). The results were not similar for the mean-pairwise distance (MPD); the MPD was largely independent of the species richness of an assemblage (Fig. 4E). Functional similarities based on mean neighbor pairwise distances and functional Sorensen's were generally more closely related to environmental distance when geographical distance was controlled for than vice versa (Table S5). Functional similarity based on mean pairwise distances was unrelated to environmental or geographical distances, suggesting that spatial autocorrelation in community functionality was not operating on this measure in the latitudinal gradient. Taxonomic similarity based on Sorensen's index was slightly more closely related to geographical distance (Table S5). The best dbRDA model for taxonomic species turnover retained temperature annual range, PET, altitude and habitat area predictors (Adjusted R-square  = 0.36) explaining 46% of total variation. Temperature annual range, PET and altitude (Adjusted R-square  = 0.34) explained 43% of the MNTD total variation – the best dbRDA model explaining functional similarity along the gradient. For the Sorensen's functional turnover, the best dbRDA model explains 45% of the total variation and identified temperature annual range, altitude, PET and area as significant predictors (Adjusted R-square  = 0.35). However, for the mean pairwise distances (MPD), the model explained only 5% of total variation and identified temperature annual range (Adjusted R-square  = 0.013) as the single variable in the dbRDA analysis (Table S6).

### Morphological structure

**Figure 5:**
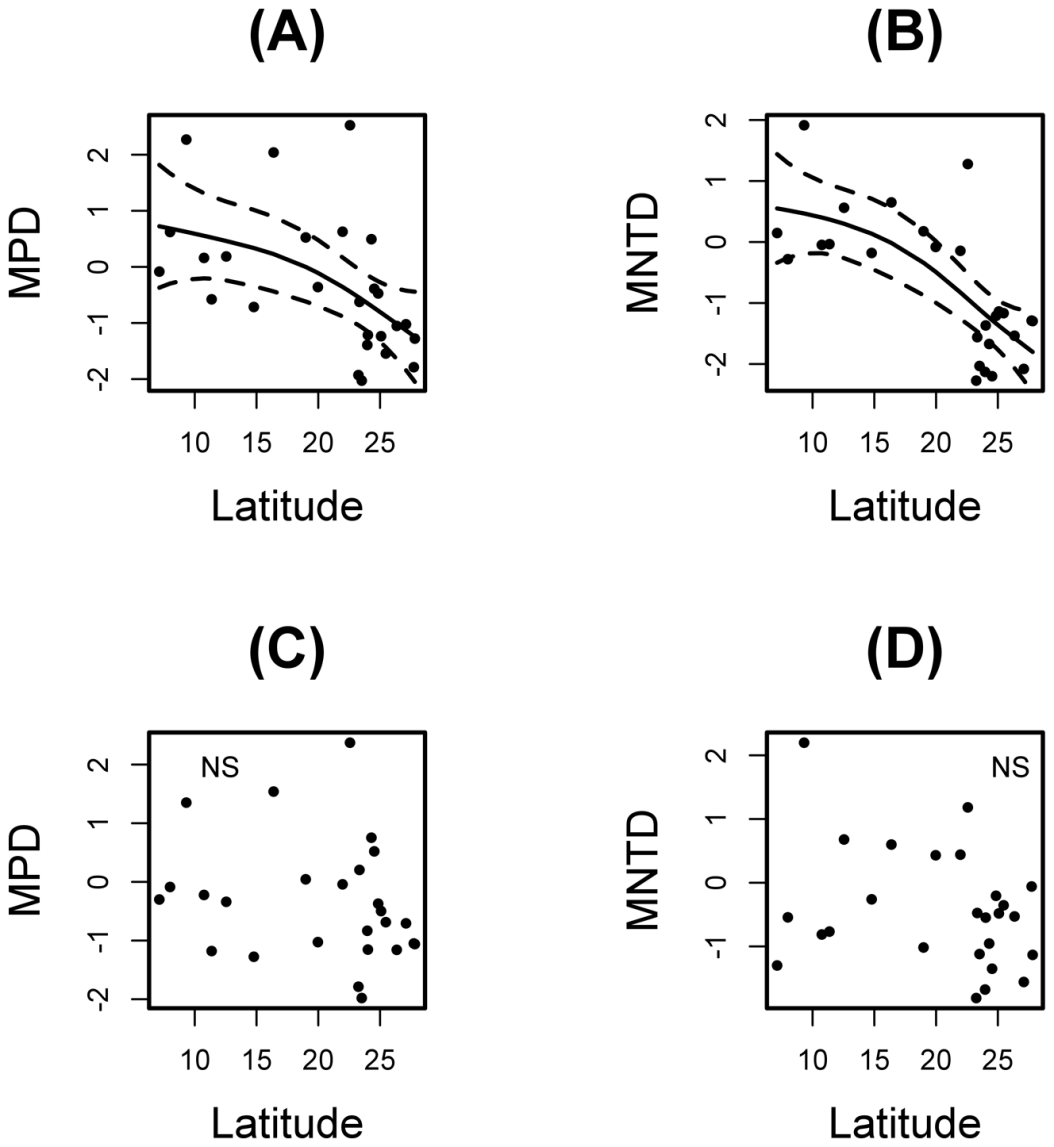
Relationship between morphological structure of the leaf-litter ant fauna along a latitudinal gradient in the Brazilian Atlantic Forest. A–B: unconstrained species pool models for MPD and MNTD, respectively; C–D: constrained species pools models for MPD and MNTD, respectively. Solid line shows the GAM model and the dashed line is the 95% confidence interval.

Overall the morphological tests support random morphological structuring along the latitudinal gradient at the community level in both delimited and unconstrained species pools. Significantly aggregated communities (SES <−1.96) occur only at higher latitudes (southern and south of the Atlantic Forest) for the unconstrained analysis (Fig. 5A–B). The number of significant results decreases in the constrained analysis (delimited species pools). For the unconstrained model, the distribution of species in the morphospace, as indicated by SES values, shifts along the gradient as showed by GAM models (30.1% and 53.6% of deviance explained by latitude in MPD and MNTD SES values, respectively); the morphological space of communities shifts from overdispersed (North) to aggregated (South) along the gradient (Fig. 5A–B). However, community morphospace does not relate to latitude for SES values in the constrained analysis (i.e., species pool defined for regions in the Atlantic Forest; Fig. 5C–D; Table S7).

We recognize nine morphological guilds in the Atlantic Forest leaf-litter ant fauna [28]. The generalist species compose the richest guild (131 species). Large and medium-size epigaeic predators are the species guilds with lowest richness (21 species each). Atlantic Forest leaf-litter ant guilds show similar patterns in species range distributions along the ecosystem; on average, 43% of species guilds have distribution covering more than 500 km, while a larger number of species display more limited distributions (Fig. S1), suggesting relatively high within-guild species turnover along the gradient.

Within-guilds morphological structure was in general random. When morphological structure was detected (Table S8), the space was aggregated in both models (unconstrained and constrained by species pools). Moving from lower to higher latitudes, the morphological space of guilds in the unconstrained model shifts from overdispersed to aggregated in fungus-growers, generalist species and medium-size epigaeic predators, as determined by GAM models (Figs. S2, S3). In the constrained model, the same response was detected for fungus-growers, small-size hypogaeic generalists and small-size hypogaeic predators (Figs. S4, S5); on the other hand, the medium-size epigaeic predators shows an inverse pattern (from aggregate to overdispersed from lower to higher latitudes) (Fig. S4).

## Discussion

Community ecologists are increasingly using quantitative diversity components of assemblages in an integrative approach encompassing taxonomic, phylogenetic and functional diversity dimensions [50], [51], [52], [53]. Understanding spatial patterns of functional diversity at relatively big scales can be a useful approach to disentangle the relative importance of assembly processes [54]. Further, large-scale studies on biodiversity along environmental gradients are essential to estimate functional species pools and to detect patterns of trait diversity [45].

### Species richness and functional diversity across the Atlantic Forest

Our results show that facets of biodiversity respond differently to a tropical forest latitudinal gradient. Leaf-litter ant species richness (number of species in each locality) showed an inverse relationship with latitude, even after adjusted for the number of individuals. The functional specialization of the ant leaf-litter communities showed a classical linear response to the latitudinal gradient, increasing from high to low latitudes, defined as the average distance of its species to the center of gravity in the morphological space. In this case, species size, eye position and body shape change on average faster at low than in high latitudes in the Atlantic Forest, leading to communities made up of the same groups of species, although more functionally distinct at low latitudes. Functional richness (defined by two metrics) and functional evenness either were not or were weakly related to the latitudinal gradient, suggesting that the amount of functional space occupied by the assemblages and the regularity of the species abundances distribution in the morphological space [55] do not change significantly along the gradient. The functional divergence, expressing how far high species abundance stands from the center of the morphological space [15] showed positive relationships with latitude but negative relationship with altitude. Together, our results suggest a contrast between the positive response of species richness to the gradient and the negative response of functional specialization. Further, our results imply that, although there is a high species turnover as a function of the environmental distances along the Atlantic forest, the functional structure of assemblages (complementary and guild structure) remains unchanged along the gradient.

The observed inverse latitudinal gradient of ant species richness is intriguing; although the observed pattern is supported even accounting for species occurrences or when high altitude areas were excluded from models. We hypothesized that the increase in leaf-litter ant species richness with latitude in the Atlantic Forest may reflect the synergy of two processes affecting spatial turnover. First, environmental heterogeneity may significantly influence community structure [56], a hypothesis supported by differences in species pools along the latitudinal gradient; high and low altitude areas (south and southeastern) showed larger species pool (summed) than mid-latitude or northeastern areas. The southeastern and south of the coastal Atlantic forest have higher environmental heterogeneity along the 1500 km system of mountain ranges, which altitudes are between sea level up to 2,900 meters [26]. Further, allowing for heterogeneity in our models provided more effective results for the relationship between latitude and taxonomic or morphological diversity. In our data set, species composition is also influenced by elevation. Indeed, southeastern submontane areas (>700 m) separated by as long as 600 km are more similar between them than paired low (<100 m) and submontane south areas separated by only 9 km, although close localities show more similar environmental profiles than with distant localities. Second, the Atlantic Forest is characterized by an inverse gradient in productivity, increasing the primary productivity in higher latitudes as indicated by annual actual evapotranspiration data (see Table S1). The higher rainfall in the southern part of the biome, determined by the Atlantic flank of the mountain ranges, shapes rich evergreen tropical broadleaf forests at higher latitudes. Vegetation with high regional species diversity characterizes subtropical wet forests (5,000–6,000 species at Rio de Janeiro mountain ranges) [57].

The cumulative effects of human activities along 500 years of Atlantic Forest occupation have led to a vast lowland habitat loss and hyper fragmentation in the northeastern of the Atlantic Forest [58], [59]. However, because the multiple-predictor models consistently exclude habitat area as an important variable in the models, the diversity gradient seems not to be confounded by the fragmentation gradient, given the gathered information. Further, if fragmentation is an indicator of gradient patterns in species diversity, the functional space of communities (volume or range) has not been altered after 500 years of perturbations in the Atlantic Forest, although species are being lost at specific guilds. A study [60] in an Argentinean plant community suggested that 75% of the species could be lost by random extinction before detection of a decrease in functional group richness. Further, the functional specialization gradient suggests that there are functionally distinct species coexisting at low species richness sites and potentially more functional redundancy at higher species richness sites. In this case, as richness increases, species are packed into the morphospace, as opposed to the expanding morphospace as species are added. We suggest that functional redundancy at high latitudes may be explained by the inverse latitudinal productivity gradient in the Atlantic Forest.

### Functional and taxonomic beta diversities across the Atlantic Forest

Community turnover along a spatial gradient is an increasingly recognized biogeographic pattern known as distance decay in communities' similarity studies [61], [62]. Distance decay refers to a decrease in among-site measures of similarity that corresponds to increasing geographic distance. Distance-decay patterns could be driven by differences in environmental conditions across space and by neutral models (dispersal-limitation, ecological and evolutionary drift) [63]. However, the congruence between taxonomic and functional turnovers at larger scales is less well understood, as well as the importance of deterministic and stochastic processes [64]. We found a strong influence of the environmental distance between sites in predicting among-site variation in taxonomic and functional composition. This was especially true for taxonomic similarity and consequently for the average functional similarity between nearest neighbors in the communities and on shared function between communities (functional Sorensen's index). The relationship between turnover (taxonomic or functional) and latitude was mostly governed by variables commonly identified in global models that explain ant species richness (temperature, a proxy to energy input (PET) and altitude) [36].

### Ant morphological structure across the Atlantic Forest

Our results show random patterns in morphology at community-level, suggesting that niche processes do not influence community assembly by acting on the measured traits. When communities are morphologically structured, we found that the most prevalent pattern is aggregation. We found weak support for niche-structured communities because at regional or local scales morphology was not different from random. Further, upon closer examination of trait dissimilarity constrained by Atlantic Forest regions, we did not find consistent patterns of trait diversity related to biotic processes. The unconstrained species pools analysis on the relationship between latitude and SES values (indicating the distribution of species within the morphospace), as shown in Fig. 5A–B, strongly suggests a transition from assembly processes causing trait divergence in species-poor low latitude communities to assembly processes causing trait convergence in species-rich, high latitude communities. Shifts in community structure along habitat gradients have been reported to Floridian oaks [65], alpine plants [66], and tropical hummingbirds [67], but have not been yet reported for hyperdiverse terrestrial invertebrates.

The use of guild classification based on morphology [28] provided a powerful and promising tool for describing patterns or to detect shifts in the morphospace along environmental gradients. How morphological structure relates to latitude may be determined by ant biology. In the present study, while for medium-size and small-size hypogaeic predators and generalist species, the morphospace shifts from overdispersed to aggregated from lower to higher latitudes, large-size epigaeic predators show an inverse pattern (aggregated to overdispersed space). Our models suggest more species coexisting at higher latitudes communities and also at within-guilds than expected (lower than the expected morphological dissimilarity). Studies showed that regional phylogenetic clustering increases with latitude [68], [69] in temperate areas, linked to the conservation of cold tolerance across the phylogeny [68]. Morphological aggregation is commonly correlated with environmental filtering, which reflects the assemblage of species with shared ecological tolerances resulting in clustered phenotypes in trait space [24], [70]; trait-environment links have been described for ant communities [71], [72], [73], [74]. Our results imply that the inverse latitudinal gradient in species richness is mainly driven by morphological packing within-guilds, because the volume of the morphological space does not shift along the gradient.

We have used morphological traits with known ecological function in generalist and specialist ant guilds [28]. Our results show that morphological traits that define ant guilds may be important to determine in which species groups morphological shifts occur along environment gradients. On the other hand, we are aware of the limitations of our morphological traits to fully characterize ant function in a community and that other traits may drive ecological functions. Therefore, we suggest also that other potentially important species-level data should be pursued in order to provide a better description of the functional space, specially, nest site and diet preferences, colonial social structure, and physiological and metabolism related traits [75].

## Conclusions

Our dataset is unique in focusing an hyperdiverse invertebrate taxon, in covering a large extension in one of the world's most species-rich tropical rainforests and by combining complementary aspects of community structure, as compositional, functional and morphological data in a hotspot of biodiversity [28], [71]. We have shown that splitting components of diversity is an appropriate approach to elucidate processes determining community assembly [76] and may have important consequences in understanding biotic communities occupying environmental gradients. The most important conclusion is that a framework that integrates information based on taxonomic, functional traits, and morphology data covering large scales (sample, site and regional) should be considered to understand the mechanisms involved in shaping communities ecology. A much higher precision in predicting biodiversity patterns can be reached combining different components of hyperdiverse taxa community structure than by using traditional variables. We suggest also that for invertebrate community ecology, a functional framework based on flexible enough traits should be pursued to allow comparisons at local, regional and global levels.

## Supporting Information

Figure S1 **Relationship between species richness and geographic range for each leaf-litter ant guild along the Brazilian Atlantic Forest.** LEP =  Large-size epigaeic predators; MEP =  medium-size epigaeic predators; MHP =  medium-size hypogaeic predators; SHP =  small-size hypogaeic predators; SP =  specialized predators; DP =  Dacetine predators; G =  generalists species; SHG =  small-size hypogaeic generalists; FG =  fungus-growers.
(TIFF)

Figure S2 **Relationship between morphological structure (MPD) for leaf-litter ant guilds and latitude along the Brazilian Atlantic Forest (unconstrained species pool).** Guild codes like in Fig. S1. The MPD SES values were calculated using an unconstrained species pool (randomization across the Atlantic Forest species pool). The solid lines were fitted with GAM models and dashed lines denote the 95% pointwise confidence intervals of the GAM estimate.
(TIFF)

Figure S3 **Relationship between morphological structure (MNTD) for leaf-litter ant guilds and latitude along the Brazilian Atlantic Forest (unconstrained species pool).** Guild codes like in Fig. S1. The MNTD SES values were calculated using an unconstrained species pool (randomization across the Atlantic Forest species pool). The solid lines were fitted with GAM models and dashed lines denote the 95% pointwise confidence intervals of the GAM estimate.
(TIFF)

Figure S4 **Relationship between morphological structure (MPD) for leaf-litter ant guilds and latitude along the Brazilian Atlantic Forest (constrained species pool).** Guild codes like in Fig. S1. The MPD SES values were calculated using a constrained species pools (randomization across regional pools for each Atlantic Forest region). The solid lines were fitted with GAM models and dashed lines denote the 95% pointwise confidence intervals of the GAM estimate.
(TIFF)

Figure S5 **Relationship between morphological structure (MNTD) for leaf-litter ant guilds and latitude along the Brazilian Atlantic Forest (constrained species pool).** Guild codes like that in Fig. S1. The MNTD SES values were calculated using a constrained species pools (randomization across regional pools for each Atlantic Forest region). The solid lines were fitted with GAM models and dashed lines denote the 95% pointwise confidence intervals of the GAM estimate.
(TIFF)

Table S1 **Atlantic Forest leaf-litter ant sampling sites, coordinates, site characteristics (habitat area and altitude) and environmental parameters employed in the analyses.** Lat =  Latitude; Long  =  Longitude; Area =  area (hectare); Alt  =  altitude (elevation above sea level); Prec  =  annual precipitation; MinT  =  minimum temperature (the average minimum temperature of the coldest month); MaxT  =  maximum temperature (the average maximum temperature of the warmest month); Trange  =  temperature annual range; Tmean  =  annual mean temperature; AET  =  annual actual evapotranspiration; PET  =  annual potential evapotranspiration.
(PDF)

Table S2 **Leaf-litter ant species registered in the Brazilian Atlantic Forest, guild classification and morphological traits used in this study.** *Guilds*: LEP =  Large-size epigaeic predators; MEP =  medium-size epigaeic predators; MHP =  medium-size hypogaeic predators; SHP =  small-size hypogaeic predators; SP =  specialized predators; DP =  Dacetine predators; G =  generalists species; SHG =  small-size hypogaeic generalists; FG =  fungus-growers; N =  army-ants; V =  arboricolous ants. *Morphological traits*: AL =  mesosoma length; PeL =  petiole length; PeH =  petiole height; FL =  hind femur length; MW =  mandible width; ID =  interocular distance; CL =  clypeus length; EL =  compound eye length; DEM =  distance of compound eye to mandible insertion.
(PDF)

Table S3 (**A**) Generalized Additive Model (GAM) results for the leaf-litter ant taxonomic and functional diversity in 26 Atlantic Forest sites. The predictors are smoothers for Latitude and Altitude. e.d.f., Estimated degrees of freedom. (**B**) Generalized Linear Model (GLM) results for the leaf-litter ant diversity (taxonomic) in low land Atlantic Forest sites (<400 m). The predictors are Latitude and Habitat Area. d.f., Degrees of freedom.
(PDF)

Table S4 (**A**) Multiple Predictor Models of taxonomic and functional diversities (Model AIC values) for the leaf-litter ant fauna in low (<400 m) and high (>700 m) land Atlantic forest areas. Predictors are Temperature Annual Range, Altitude (m), AET, Precipitation and Habitat Area. Values in bold indicates significant variable contributing to the model (** 0.01, *0.05). (**B**) Multiple Predictor Models of taxonomic and functional diversities (Model AIC values) for the leaf-litter ant fauna in low land Atlantic forest areas (<400 m). Predictors are Temperature Annual Range, Altitude (m), AET, Precipitation and Habitat Area. Values in bold indicates significant variable contributing to the model (*** 0.001, ** 0.01, *0.05, #0.1).
(PDF)

Table S5 **Partial Mantel correlations between community or functional similarity and environmental distance controlling for geographic distance (and vice versa).**
(PDF)

Table S6 **Summary of model selection on constrained ordination models (dbRDA) that describe the influence of environmental variables on the leaf-litter ant fauna along Atlantic Forest sites.**
(PDF)

Table S7 **Summary of GAMs to examine the relationship between the morphological structure of the leaf-litter ant assemblages and latitude in the Atlantic Forest.**
(PDF)

Table S8 **Summary of GAMs to examine the relationship between the morphological structure of the leaf-litter ant guilds and latitude in the Atlantic Forest.**
(PDF)
