# Supplementary figures and images for: Ecosystem-Wide Morphological Structure of Leaf-Litter Ant Communities along a Tropical Latitudinal Gradient
Source: PLoS One. 2014 Mar 26;9(3):e93049. doi: 10.1371/journal.pone.0093049 (PMC3966852; doi:10.1371/journal.pone.0093049)

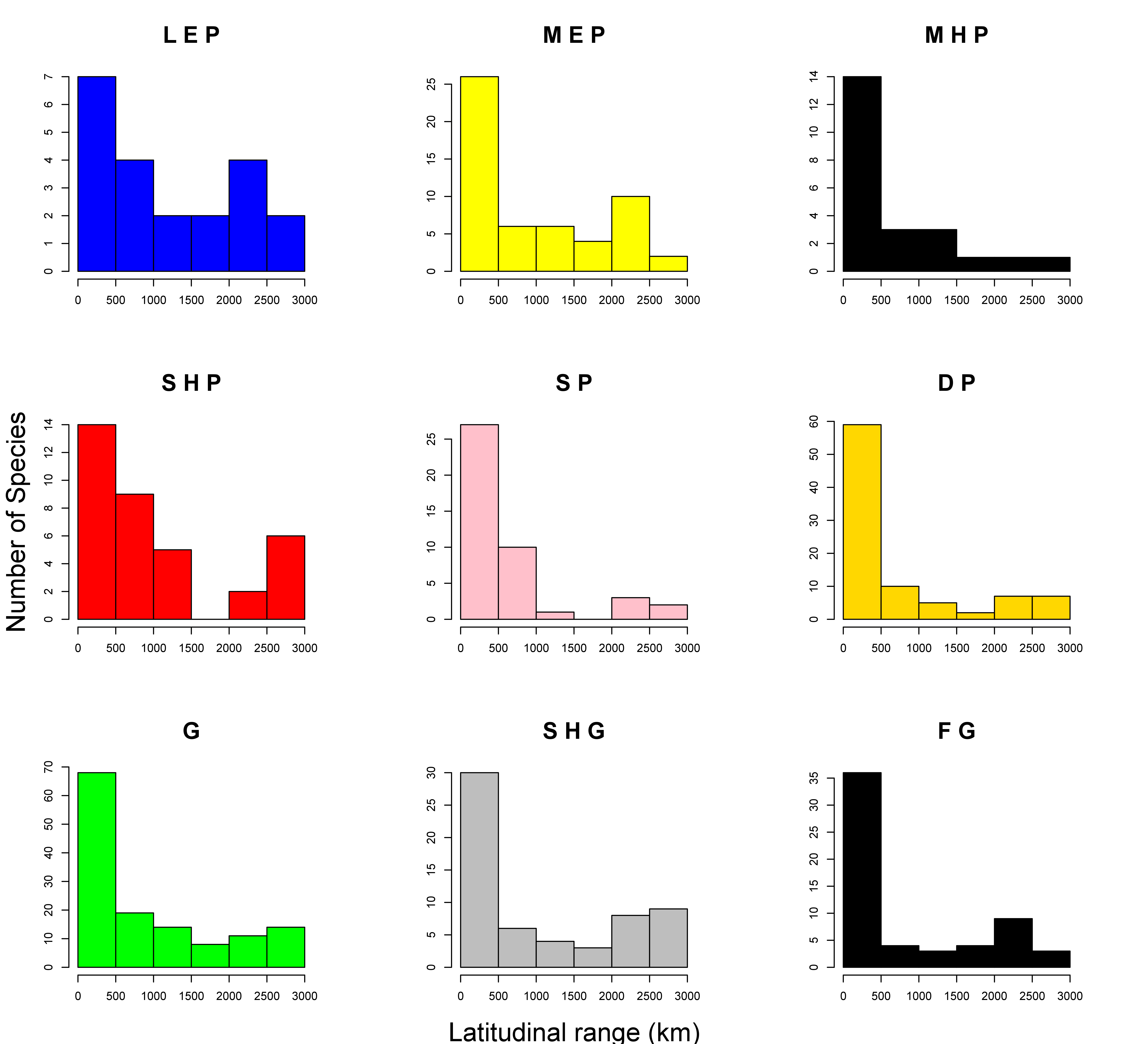

Supplement: Figure S1 — Relationship between species richness and geographic range for each leaf-litter ant guild along the Brazilian Atlantic Forest. LEP = Large-size epigaeic predators; MEP = medium-size epigaeic predators; MHP = medium-size hypogaeic predators; SHP = small-size hypogaeic predators; SP = specialized predators; DP = Dacetine predators; G = generalists species; SHG = small-size hypogaeic generalists; FG = fungus-growers. (TIFF) [file pone.0093049.s001.tiff]

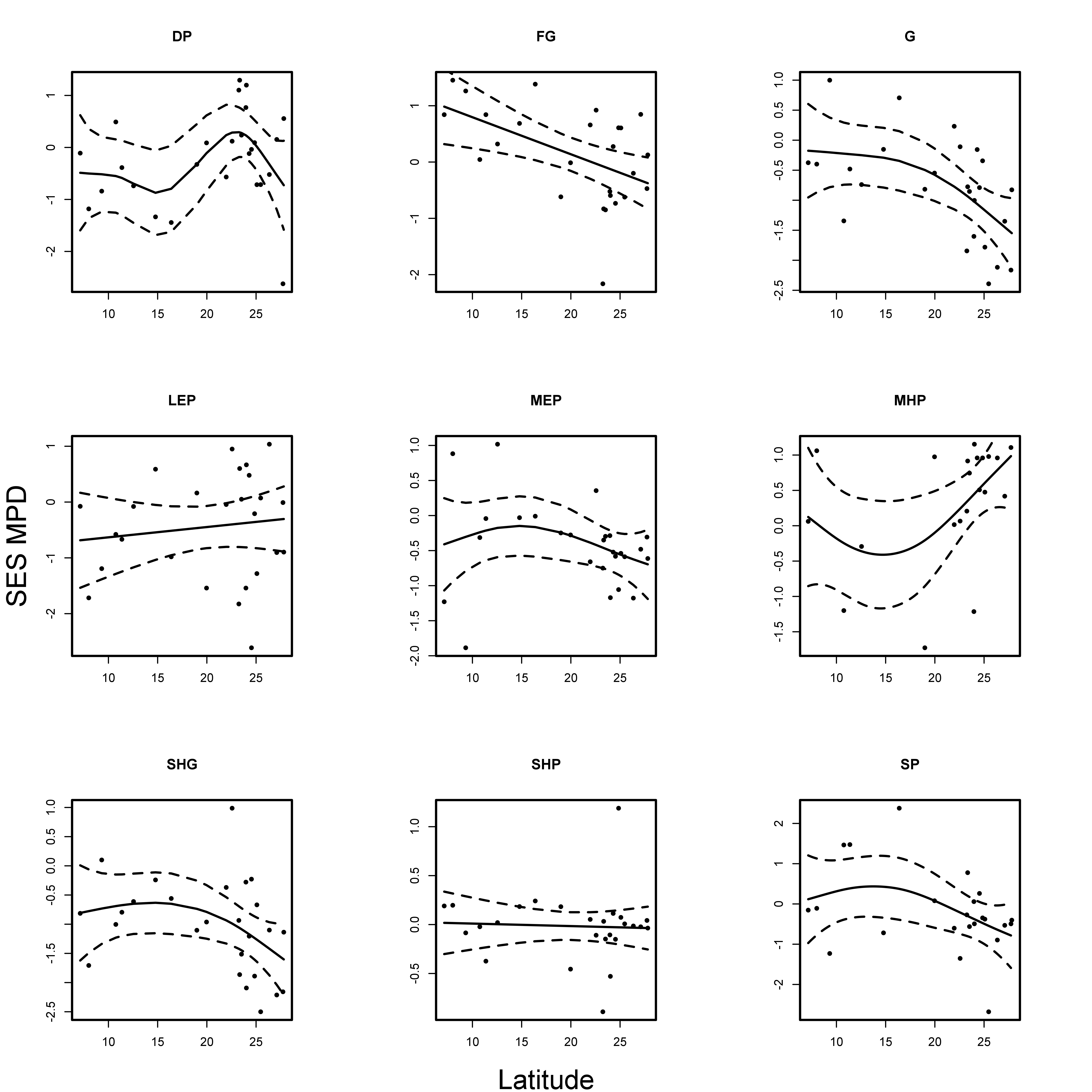

Supplement: Figure S2 — Relationship between morphological structure (MPD) for leaf-litter ant guilds and latitude along the Brazilian Atlantic Forest (unconstrained species pool). Guild codes like in Fig. S1. The MPD SES values were calculated using an unconstrained species pool (randomization across the Atlantic Forest species pool). The solid lines were fitted with GAM models and dashed lines denote the 95% pointwise confidence intervals of the GAM estimate. (TIFF) [file pone.0093049.s002.tiff]

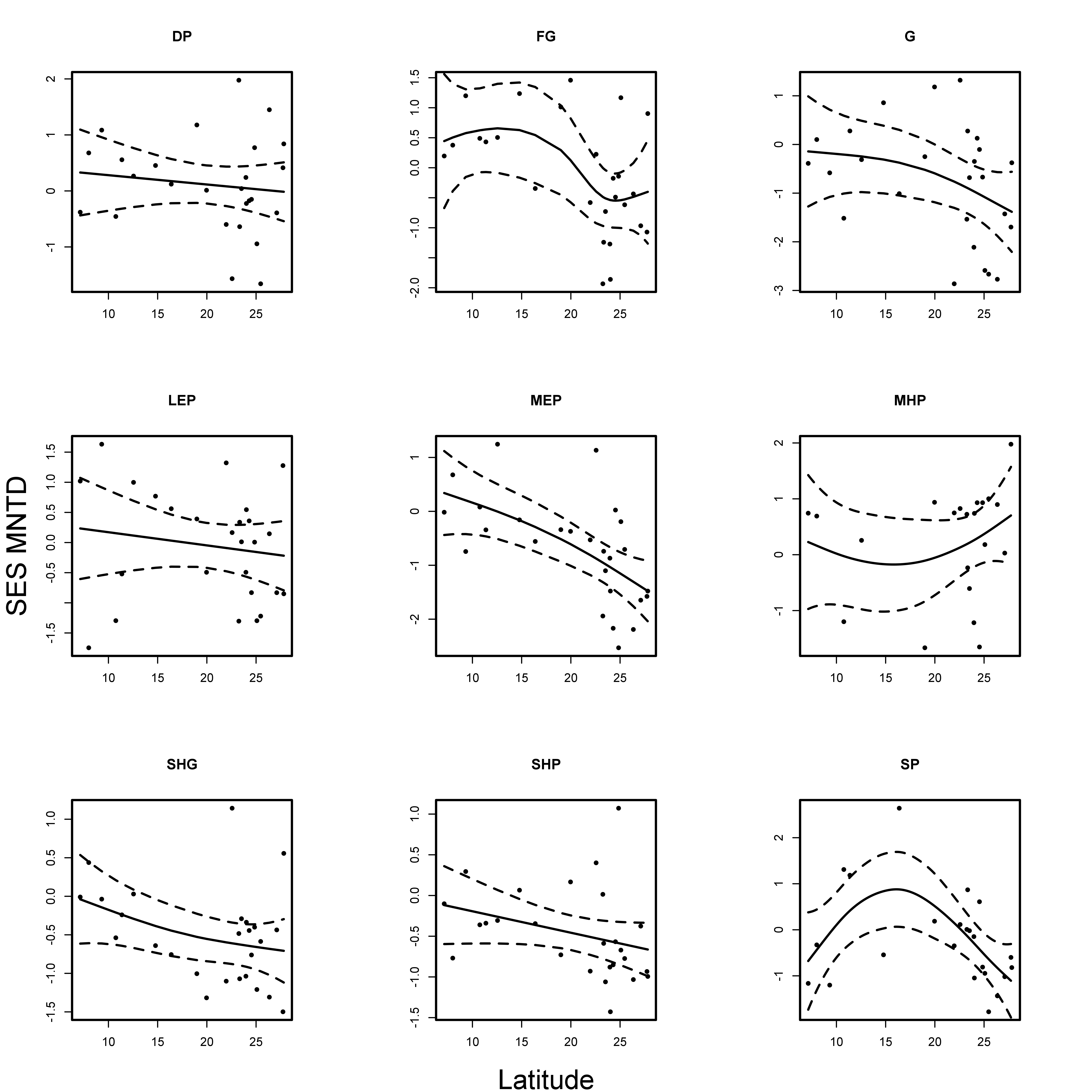

Supplement: Figure S3 — Relationship between morphological structure (MNTD) for leaf-litter ant guilds and latitude along the Brazilian Atlantic Forest (unconstrained species pool). Guild codes like in Fig. S1. The MNTD SES values were calculated using an unconstrained species pool (randomization across the Atlantic Forest species pool). The solid lines were fitted with GAM models and dashed lines denote the 95% pointwise confidence intervals of the GAM estimate. (TIFF) [file pone.0093049.s003.tiff]

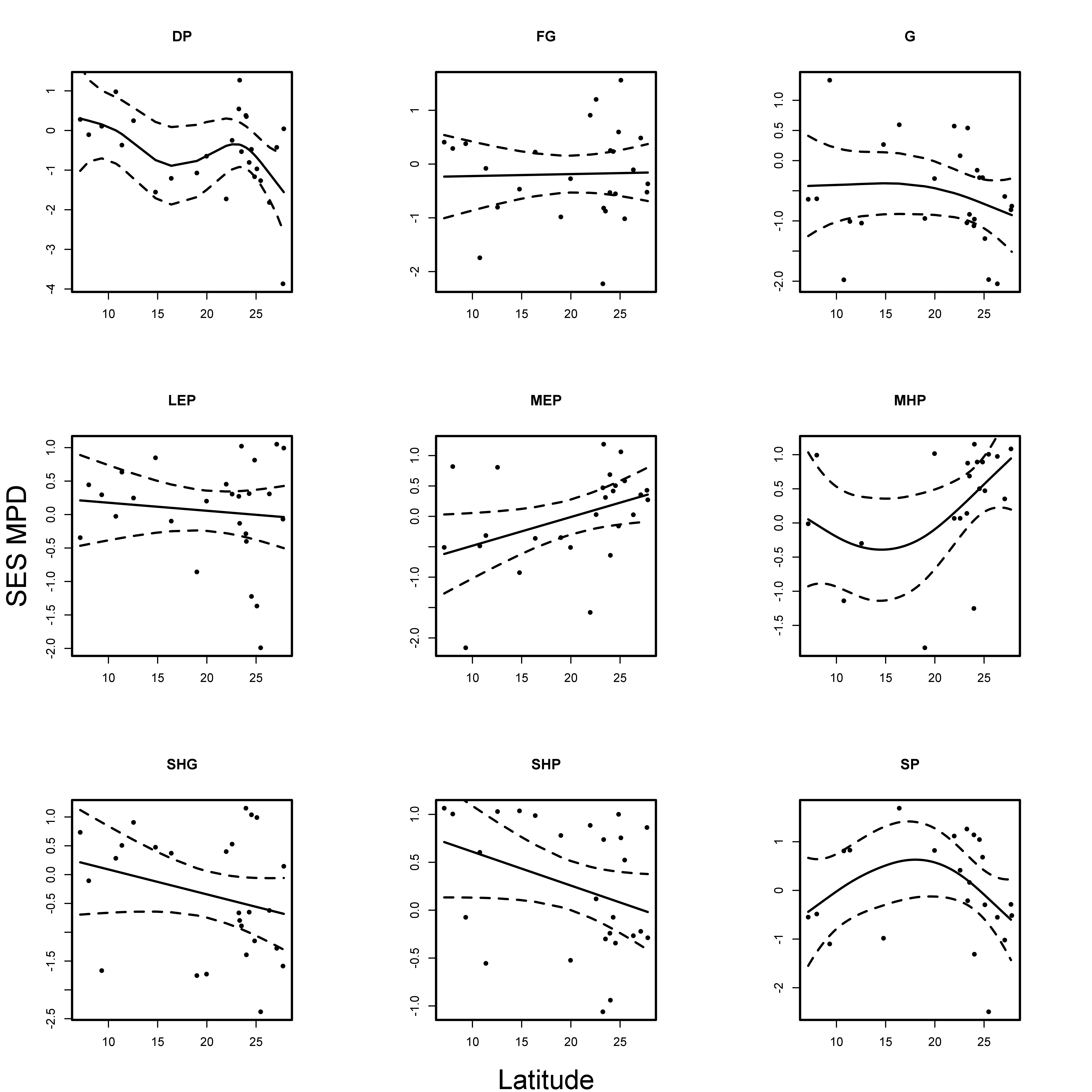

Supplement: Figure S4 — Relationship between morphological structure (MPD) for leaf-litter ant guilds and latitude along the Brazilian Atlantic Forest (constrained species pool). Guild codes like in Fig. S1. The MPD SES values were calculated using a constrained species pools (randomization across regional pools for each Atlantic Forest region). The solid lines were fitted with GAM models and dashed lines denote the 95% pointwise confidence intervals of the GAM estimate. (TIFF) [file pone.0093049.s004.tiff]

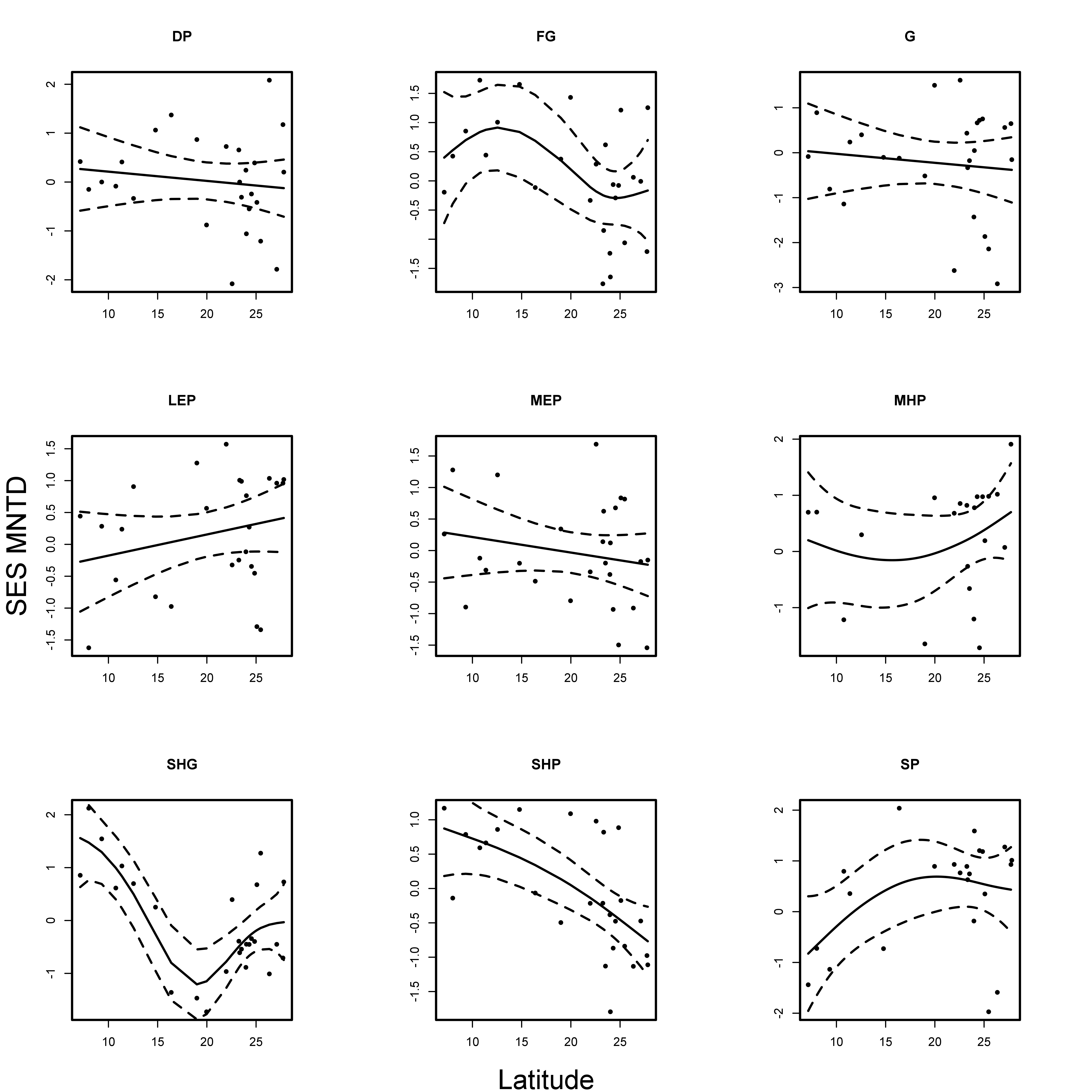

Supplement: Figure S5 — Relationship between morphological structure (MNTD) for leaf-litter ant guilds and latitude along the Brazilian Atlantic Forest (constrained species pool). Guild codes like that in Fig. S1. The MNTD SES values were calculated using a constrained species pools (randomization across regional pools for each Atlantic Forest region). The solid lines were fitted with GAM models and dashed lines denote the 95% pointwise confidence intervals of the GAM estimate. (TIFF) [file pone.0093049.s005.tiff]
