# Supplementary material for: Ecosystem-Wide Morphological Structure of Leaf-Litter Ant Communities along a Tropical Latitudinal Gradient
Source: PLoS One. 2014 Mar 26;9(3):e93049. doi: 10.1371/journal.pone.0093049 (PMC3966852; doi:10.1371/journal.pone.0093049)
Supplement: Table S1 — Atlantic Forest leaf-litter ant sampling sites, coordinates, site characteristics (habitat area and altitude) and environmental parameters employed in the analyses. Lat = Latitude; Long = Longitude; Area = area (hectare); Alt = altitude (elevation above sea level); Prec = annual precipitation; MinT = minimum temperature (the average minimum temperature of the coldest month); MaxT = maximum temperature (the average maximum temperature of the warmest month); Trange = temperature annual range; Tmean = annual mean temperature; AET = annual actual evapotranspiration; PET = annual potential evapotranspiration. (PDF) [file pone.0093049.s006.pdf]

Table S1. Atlantic Forest leaf-litter ant sampling sites, coordinates, site characteristics (habitat area and altitude) and environmental parameters employed in the analyses. Lat= Latitude; Long = Longitude; Area= area (hectare); Alt = altitude (m); Prec = annual precipitation; MinT = minimum temperature (the average minimum temperature of the coldest month); MaxT = maximum temperature (the average maximum temperature of the warmest month); Trange = temperature annual range; Tmean = annual mean temperature; AET = annual actual evapotranspiration; PET = annual potential evapotranspiration.

Atlantic Forest sites	Lat	Long	Area	Alt	Prec	MinT	MaxT	Trange	Tmean	PET	AET
Mata do Buraquinho	-34.859	-7.140	515	36	1929	19.2	30.7	11.5	25.5	1483	1130
Horto Dois Irmãos	-34.944	-8.009	338	41	1768	20.7	30.3	9.6	25.6	1385	1043
Quebrangulo	-36.476	-9.322	4636	359	1432	17	30.3	13.3	23.2	1521	976
Parque Estadual da Serra de Itabaiana	-37.333	-10.765	4300	194	1277	18.9	29.7	10.8	24.3	1383	936
Crasto	-37.419	-11.378	900	19	1671	19.3	31.5	12.2	25.2	1523	1116
Reserva Sapiranga	-38.511	-12.971	600	20	1833	20.5	30.6	10.1	25.3	1344	1134
Mata da Boa Esperança	-39.066	-14.796	437	23	1901	19.4	29.6	10.2	24.7	1284	1277
Estação Ecológica Pau Brasil	-39.183	-16.393	1151	94	1474	18	29.5	11.5	24	1386	1201
REBIO Sooretama	-39.949	-19.073	27946	47	1226	17	31.2	14.2	24	1490	1037
Estação Biológica Santa Lúcia	-40.538	-19.969	440	766	1303	12.7	28.1	15.4	20.4	1409	1104
Parque Estadual do Desengano	-41.950	-21.969	22400	928	1452	8.7	25.3	16.6	17.4	1300	1138
REBIO Tinguá	-43.414	-22.571	26000	261	2031	13.1	30.1	17	21.6	1481	1385
Parque Estadual Serra do Mar, Núcleo Cunha-Indaiá	-45.007	-23.251	14000	1082	1664	5.7	23.8	18.1	15.9	1286	1137
Parque Estadual Serra do Mar, Núcleo Picinguaba	-44.838	-23.330	7850	288	2302	12.4	29.7	17.3	21.7	1438	1406
Estação Biológica Boracéia	-45.846	-23.532	96	810	1609	8.4	24.8	16.4	17.4	1236	1151
Parque Estadual Serra do Mar, Núcleo Pilões-Cubatão	-46.540	-23.975	115000	188	2709	13.7	28.3	14.6	21.3	1230	1230
Parque Estadual Serra do Mar, Tapiraí	-47.466	-24.032	6000	320	1621	11	28.1	17.1	20.1	1353	1249
Parque Estadual Intervales, Base Barra Grande	-48.365	-24.305	41705	857	1416	7.6	26.3	18.7	17.5	1295	1163
Estação Ecológica Juréia-Itatins, Núcleo Rio Verde	-47.236	-24.544	79270	167	2001	12.7	29	16.3	21.2	1307	1295
Parque Estadual das Lauráceas	-48.717	-24.854	27524	856	1447	8.2	26.3	18.1	17.7	1253	1194
Parque Estadual Ilha do Cardoso-SP	-47.930	-25.097	22500	22	2490	13.8	30.9	17.1	22.5	1350	1348
Parque Estadual do Pau-Oco	-48.889	-25.576	905	263	1896	10.9	28.9	18	20.1	1275	1265
APA Rio Vermelho	-49.271	-26.364	23000	298	1529	10.5	28.9	18.4	19.6	1328	1265
Parque Estadual Nascentes	-49.154	-27.104	5300	794	1662	7.7	25.7	18	16.6	1201	1182
Parque Estadual Serra do Tabuleiro	-48.697	-27.741	87405	298	1584	10.7	26.4	15.7	18.5	1073	1064
Parque Estadual Serra do Tabuleiro	-48.911	-27.818	87405	798	1681	7.7	24.5	16.8	16	1101	1093
