# Supplementary material for: Ecosystem-Wide Morphological Structure of Leaf-Litter Ant Communities along a Tropical Latitudinal Gradient
Source: PLoS One. 2014 Mar 26;9(3):e93049. doi: 10.1371/journal.pone.0093049 (PMC3966852; doi:10.1371/journal.pone.0093049)
Supplement: Table S2 — Leaf-litter ant species registered in the Brazilian Atlantic Forest, guild classification and morphological traits used in this study. Guilds: LEP = Large-size epigaeic predators; MEP = medium-size epigaeic predators; MHP = medium-size hypogaeic predators; SHP = small-size hypogaeic predators; SP = specialized predators; DP = Dacetine predators; G = generalists species; SHG = small-size hypogaeic generalists; FG = fungus-growers; N = army-ants; V = arboricolous ants. Morphological traits: AL = mesosoma length; PeL = petiole length; PeH = petiole height; FL = hind femur length; MW = mandible width; ID = interocular distance; CL = clypeus length; EL = compound eye length; DEM = distance of compound eye to mandible insertion. (PDF) [file pone.0093049.s007.pdf]

Table S2. Leaf-litter ant species registered in the Brazilian Atlantic Forest, guild classification and morphological traits used in this study. *Guilds*: LEP= Large-size epigaeic predators; MEP= medium-size epigaeic predators; MHP= medium-size hypogaeic predators; SHP= small-size hypogaeic predators; SP= specialized predators; DP= Dacetine predators; G= generalists species; SHG= small-size hypogaeic generalists; FG= fungus-growers; N= army-ants; V= arboricolous ants.
Morphological traits: AL= mesosoma length; PeL= petiole length; PeH= petiole height; FL= hind femur length; MW= mandible width; ID= interocular distance; CL= clypeus length; EL= compound eye length; DEM= distance of compound eye to mandible insertion.

Species	Guild	AL	PeL	PeH	FL	MW	ID	CL	EL	DEM
Acanthognathus brevicornis	DP	0.811	0.507	0.185	0.696	0.067	0.493	0.248	0.156	0.382
Acanthognathus ocellatus	DP	0.756	0.448	0.167	0.674	0.061	0.452	0.215	0.119	0.348
Acanthognathus rudis	DP	0.796	0.459	0.178	0.715	0.07	0.452	0.237	0.126	0.374
Acanthoponera mucronata	V	2.571	0.771	0.857	1.571	0.486	1.286	0.6	0.371	0.714
Acanthosticus sp.	SP	1.367	0.531	0.326	0.688	0.302	0.759	0.074	0.019	0.438
Acromyrmex aspersus	FG	1.771	0.309	0.257	2.034	0.377	1.223	0.394	0.217	0.286
Acromyrmex crassispinus	FG	1.811	0.326	0.274	1.874	0.383	1.274	0.349	0.197	0.246
Acromyrmex disciger	FG	1.979	0.343	0.3	2.179	0.4	1.336	0.336	0.236	0.271
Acromyrmex niger	FG	1.943	0.32	0.269	2.177	0.371	1.269	0.377	0.2	0.291
Acropyga decendens	G	0.648	0.148	0.227	0.456	0.137	0.504	0.152	0.035	0.137
Acropyga exsanguis	G	0.444	0.093	0.167	0.352	0.093	0.352	0.111	0.019	0.093
Acropyga fuhrmanni	G	0.548	0.122	0.185	0.378	0.115	0.43	0.152	0.028	0.115
Acropyga goeldii	G	0.574	0.12	0.185	0.393	0.096	0.444	0.115	0.026	0.122
Acropyga guianensis	G	0.458	0.116	0.162	0.259	0.097	0.31	0.106	0.019	0.102
Acropyga panamensis	G	0.482	0.111	0.148	0.259	0.074	0.315	0.093	0.019	0.093
Acropyga smithii	G	0.463	0.102	0.162	0.333	0.1	0.352	0.104	0.024	0.093
Adelomyrmex sp.1	SP	0.71	0.309	0.173	0.475	0.123	0.506	0.154	0.037	0.179
Adelomyrmex sp.2	SP	0.611	0.296	0.148	0.426	0.13	0.463	0.13	0.019	0.167
Anochetus (Gr. Inermis) sp.1	LEP	0.837	0.148	0.204	0.57	0.085	0.452	0.104	0.074	0.244
Anochetus altisquamis	LEP	1.1	0.2	0.33	0.763	0.152	0.693	0.133	0.107	0.278
Anochetus mayri	LEP	0.744	0.119	0.182	0.511	0.082	0.426	0.111	0.07	0.233
Anochetus simoni	LEP	0.985	0.174	0.233	0.704	0.093	0.437	0.122	0.096	0.252
Anochetus sp.1	LEP	0.556	0.093	0.148	0.37	0.056	0.315	0.093	0.037	0.185
Anochetus sp.2	LEP	1.083	0.194	0.222	0.833	0.093	0.426	0.148	0.111	0.25
Apterostigma acre	FG	1.278	0.25	0.185	1.13	0.13	0.565	0.278	0.139	0.306
Apterostigma sp.1	FG	1.485	0.367	0.207	1.359	0.185	0.641	0.255	0.174	0.344
Apterostigma sp.2	FG	1.541	0.341	0.23	1.407	0.17	0.644	0.178	0.189	0.33
Apterostigma sp.3	FG	1.426	0.296	0.215	1.304	0.156	0.626	0.167	0.159	0.337
Apterostigma sp.4	FG	1.333	0.296	0.211	1.182	0.17	0.567	0.248	0.178	0.293
Apterostigma sp.5	FG	2.019	0.504	0.263	2.037	0.233	0.804	0.3	0.233	0.407
Apterostigma sp.6	FG	1.426	0.269	0.231	1.389	0.157	0.593	0.324	0.176	0.333
Azteca sp.	G	0.833	0.074	0.148	0.778	0.204	0.5	0.204	0.148	0.204

Basiceros convexiceps	MEP	1.661	0.704	0.321	1.265	0.222	0.932	0.34	0.148	0.531
Basiceros disciger	MEP	1.352	0.556	0.293	0.978	0.204	0.881	0.374	0.096	0.444
Brachymyrmex coactus	G	0.704	0.194	0.194	0.71	0.142	0.494	0.21	0.154	0.198
Brachymyrmex sp.1	G	0.396	0.093	0.111	0.352	0.063	0.285	0.1	0.07	0.1
Brachymyrmex sp.10	G	0.37	0.074	0.093	0.278	0.037	0.241	0.093	0.074	0.074
Brachymyrmex sp.11	G	0.407	0.111	0.148	0.37	0.056	0.278	0.111	0.093	0.093
Brachymyrmex sp.2	G	0.596	0.174	0.178	0.548	0.096	0.4	0.178	0.1	0.156
Brachymyrmex sp.3	G	0.459	0.117	0.142	0.381	0.07	0.315	0.122	0.082	0.119
Brachymyrmex sp.4	G	0.481	0.148	0.157	0.432	0.093	0.346	0.13	0.093	0.111
Brachymyrmex sp.5	G	0.537	0.16	0.17	0.426	0.093	0.389	0.148	0.111	0.13
Brachymyrmex sp.6	G	0.519	0.16	0.17	0.426	0.065	0.287	0.111	0.074	0.102
Brachymyrmex sp.7	G	0.534	0.154	0.159	0.479	0.079	0.378	0.156	0.085	0.148
Brachymyrmex sp.8	G	0.341	0.088	0.086	0.301	0.052	0.226	0.085	0.067	0.085
Brachymyrmex sp.9	G	0.456	0.106	0.148	0.419	0.078	0.322	0.122	0.078	0.119
Camponotus alboanulatus	G	2.11	0.404	0.473	1.661	0.355	0.853	0.506	0.4	0.686
Camponotus balzani	G	2.502	0.429	0.614	2.106	0.441	0.976	0.559	0.42	0.824
Camponotus canescens	G	1.514	0.371	0.514	1.4	0.314	0.686	0.371	0.314	0.629
Camponotus cingulatus	G	2.7	0.471	0.614	2.7	0.371	0.743	0.5	0.429	0.771
Camponotus rufipes	G	3.229	0.543	1	2.343	0.6	1.457	0.829	0.514	1.171
Camponotus sp.2	G	1.629	0.343	0.457	1.143	0.314	0.886	0.314	0.271	0.571
Camponotus sp.3	G	2.057	0.371	0.429	1.914	0.371	0.743	0.4	0.343	0.629
Camponotus sp.4	G	1.8	0.333	0.476	1.357	0.329	0.743	0.4	0.343	0.543
Camponotus sp.6	G	1.943	0.343	0.543	1.714	0.4	0.857	0.429	0.314	0.686
Camponotus trapezoideus	G	1.429	0.305	0.571	1.248	0.286	0.724	0.333	0.333	0.571
Camponotus tripartitus	G	1.8	0.343	0.543	1.286	0.4	1.086	0.429	0.286	0.686
Carebara brevipilosa	G	0.467	0.156	0.119	0.319	0.096	0.393	0.082	0.011	0.119
Carebara sp.1	SHG	0.296	0.096	0.096	0.163	0.059	0.278	0.059	0.009	0.078
Carebara sp.2	SHG	0.274	0.067	0.059	0.148	0.056	0.226	0.041	0.004	0.07
Carebara sp.3	SHG	0.282	0.078	0.063	0.152	0.048	0.215	0.048	0.003	0.093
Carebara sp.4	SHG	0.269	0.069	0.056	0.153	0.056	0.231	0.051	0.003	0.111
Carebara sp.5	SHG	0.278	0.074	0.074	0.13	0.037	0.222	0.037	0.004	0.056
Carebara sp.6	SHG	0.324	0.093	0.111	0.157	0.056	0.287	0.056	0.004	0.093
Carebara urichi	SHG	0.43	0.141	0.115	0.307	0.093	0.382	0.085	0.009	0.093
Cephalotes sp.1	G	1.222	0.204	0.278	0.278	0.204	1.222	0.259	0.259	0.685
Cephalotes sp.2	G	0.741	0.13	0.185	0.426	0.093	0.685	0.13	0.241	0.426
Cephalotes sp.3	G	1.111	0.148	0.259	0.611	0.167	0.963	0.278	0.241	0.593
Cerapachys splendens	SP	0.981	0.389	0.346	0.519	0.173	0.528	0.167	0.049	0.225
Crematogaster sp.1	G	0.763	0.333	0.159	0.748	0.185	0.515	0.215	0.144	0.226
Crematogaster sp.10	G	0.574	0.185	0.111	0.482	0.13	0.444	0.13	0.13	0.185

Crematogaster sp.2	G	0.741	0.27	0.133	0.77	0.174	0.556	0.2	0.148	0.267
Crematogaster sp.3	G	0.667	0.244	0.13	0.589	0.163	0.519	0.207	0.13	0.233
Crematogaster sp.4	G	0.722	0.204	0.13	0.528	0.204	0.648	0.204	0.139	0.232
Crematogaster sp.5	G	0.704	0.213	0.13	0.556	0.176	0.639	0.213	0.139	0.222
Crematogaster sp.6	G	0.6	0.226	0.13	0.578	0.126	0.426	0.174	0.13	0.185
Crematogaster sp.7	G	0.685	0.241	0.119	0.689	0.174	0.522	0.167	0.137	0.222
Crematogaster sp.8	G	0.733	0.289	0.152	0.707	0.182	0.556	0.211	0.144	0.233
Crematogaster sp.9	G	0.824	0.269	0.148	0.787	0.176	0.565	0.204	0.139	0.259
Cryptomyrmex boltoni	SP	0.693	0.307	0.17	0.459	0.126	0.504	0.156	0.037	0.178
Cyphomyrmex (Gr. Rimosus) sp.1	FG	0.844	0.141	0.148	0.707	0.119	0.444	0.174	0.137	0.167
Cyphomyrmex (Gr. Rimosus) sp.1(A)	FG	0.895	0.154	0.123	0.728	0.13	0.463	0.228	0.148	0.161
Cyphomyrmex (Gr. Rimosus) sp.2	FG	0.793	0.136	0.136	0.66	0.114	0.441	0.191	0.133	0.148
Cyphomyrmex (Gr. Rimosus) sp.3	FG	0.996	0.167	0.167	0.907	0.141	0.504	0.222	0.156	0.178
Cyphomyrmex (Gr. Rimosus) sp.4	FG	0.7	0.119	0.119	0.548	0.104	0.415	0.163	0.1	0.156
Cyphomyrmex (Gr. Rimosus) sp.5	FG	0.759	0.148	0.13	0.611	0.13	0.482	0.167	0.093	0.167
Cyphomyrmex (Gr. Rimosus) sp.6	FG	0.833	0.136	0.123	0.691	0.136	0.475	0.21	0.117	0.179
Cyphomyrmex (Gr. Rimosus) sp.7	FG	0.691	0.154	0.093	0.568	0.117	0.42	0.167	0.093	0.105
Cyphomyrmex (Gr. Rimosus) sp.8	FG	0.648	0.111	0.111	0.537	0.111	0.407	0.167	0.093	0.148
Cyphomyrmex (Gr. Rimosus) sp.9	FG	0.815	0.13	0.111	0.685	0.111	0.482	0.222	0.13	0.167
Cyphomyrmex (Gr. Strigatus) sp.1	FG	0.815	0.19	0.125	0.657	0.143	0.491	0.181	0.102	0.13
Cyphomyrmex (Gr. Strigatus) sp.2	FG	0.944	0.204	0.148	0.852	0.167	0.556	0.204	0.093	0.148
Cyphomyrmex (Gr. Strigatus) sp.3	FG	0.778	0.185	0.13	0.648	0.13	0.5	0.167	0.093	0.148
Cyphomyrmex (Gr. Strigatus) sp.4	FG	0.809	0.167	0.123	0.698	0.142	0.494	0.167	0.093	0.139
Cyphomyrmex (Gr. Strigatus) sp.5	FG	0.704	0.167	0.111	0.593	0.13	0.482	0.167	0.093	0.148
Cyphomyrmex (Gr. Strigatus) sp.6	FG	0.915	0.193	0.13	0.737	0.159	0.537	0.211	0.111	0.156
Cyphomyrmex (Gr. Strigatus) sp.7	FG	0.815	0.185	0.148	0.648	0.148	0.5	0.185	0.093	0.148
Cyphomyrmex strigatus	FG	1.015	0.219	0.178	0.9	0.178	0.585	0.218	0.111	0.152
Cyphomyrmex auritus	FG	1.296	0.278	0.204	1.352	0.241	0.704	0.222	0.167	0.204
Cyphomyrmex olitor	FG	0.861	0.204	0.139	0.667	0.157	0.519	0.204	0.111	0.167
Cyphomyrmex plaumanni	FG	0.937	0.193	0.17	0.778	0.156	0.537	0.19	0.104	0.156
Cyphomyrmex pr. *olitor* A	FG	0.83	0.178	0.137	0.674	0.148	0.504	0.189	0.111	0.143
Cyphomyrmex pr. *olitor* B	FG	0.726	0.163	0.104	0.578	0.119	0.441	0.17	0.089	0.122
Diaphoromyrma sofiae	G	0.463	0.176	0.222	0.38	0.148	0.435	0.167	0.13	0.056
Discothyrea neotropica	SHG	0.5	0.122	0.159	0.307	0.074	0.33	0.059	0.019	0.093
Discothyrea sexarticulata	SHG	0.396	0.096	0.141	0.222	0.085	0.326	0.059	0.006	0.122
Dolichoderus imitator	G	2.429	0.648	0.467	2.171	0.343	0.519	0.467	0.352	0.329
Dolichoderus sp.1	G	1.222	0.315	0.315	0.926	0.333	0.482	0.315	0.222	0.222
Eciton burchelli	N	2.81	0.543	0.448	3.171	0.59	1.21	0.314	0.19	0.81
Ectatomma edentatum	LEP	2.905	0.611	0.691	2.341	0.579	1.262	0.476	0.373	0.706

Ectatomma permagnum	LEP	3.81	0.857	0.952	3.143	0.762	1.619	0.619	0.667	0.905
Ectatomma tuberculatum	LEP	3.79	0.81	0.79	3.381	0.79	1.8	0.781	0.562	0.8
Eurhopalothrix gravis	DP	0.822	0.322	0.215	0.578	0.122	0.589	0.259	0.054	0.311
Eurhopalothrix sp.E	DP	0.593	0.204	0.148	0.389	0.074	0.426	0.222	0.019	0.315
Eurhopalothrix speciosa	DP	0.778	0.3	0.193	0.585	0.12	0.615	0.293	0.096	0.282
Eurhopalothrix spectabilis	DP	0.63	0.241	0.167	0.42	0.08	0.444	0.247	0.037	0.265
Gnamptogenys acuminata	SP	1.714	0.571	0.429	1.267	0.152	0.933	0.267	0.314	0.267
Gnamptogenys continua	SP	1.015	0.34	0.281	0.556	0.117	0.617	0.145	0.074	0.281
Gnamptogenys haenski	SP	2.4	0.543	0.629	1.571	0.229	1.457	0.371	0.143	0.714
Gnamptogenys horni	SP	1.095	0.357	0.3	0.705	0.11	0.629	0.181	0.143	0.305
Gnamptogenys interrupta	SP	1.614	0.529	0.414	1.014	0.214	1.043	0.3	0.129	0.443
Gnamptogenys lucaris	SP	1.695	0.476	0.371	1.238	0.152	0.886	0.257	0.238	0.371
Gnamptogenys mediatrix	MEP	1.5	0.417	0.375	0.991	0.287	0.843	0.208	0.19	0.259
Gnamptogenys minuta	SP	1.186	0.314	0.314	0.714	0.1	0.7	0.214	0.05	0.329
Gnamptogenys pleurodon	MEP	1.629	0.457	0.429	1.486	0.257	0.829	0.286	0.2	0.457
Gnamptogenys pr. *striatula* A	MEP	1.465	0.382	0.38	1.192	0.259	0.783	0.315	0.17	0.427
Gnamptogenys pr. *striatula* B	MEP	1.432	0.367	0.37	1.114	0.256	0.762	0.333	0.17	0.423
Gnamptogenys pr. *striatula* C	MEP	1.556	0.37	0.37	1.259	0.278	0.778	0.37	0.185	0.444
Gnamptogenys rastrata	MEP	1.299	0.355	0.309	0.821	0.256	0.744	0.191	0.173	0.259
Gnamptogenys reichenspergeri	SP	1.068	0.256	0.275	0.599	0.25	0.716	0.231	0.053	0.37
Gnamptogenys sp.1	SP	0.951	0.321	0.265	0.482	0.111	0.556	0.148	0.049	0.253
Gnamptogenys sp.2	SP	1.537	0.482	0.463	0.889	0.222	0.982	0.333	0.093	0.426
Gnamptogenys sp.3	SP	1.296	0.407	0.296	0.926	0.259	0.704	0.185	0.185	0.278
Gnamptogenys sp.4	SP	1.579	0.414	0.379	1.064	0.321	0.929	0.236	0.186	0.271
Gnamptogenys sp.5	SP	1.102	0.306	0.296	0.676	0.204	0.63	0.167	0.12	0.213
Gnamptogenys sp.6	SP	1.167	0.278	0.278	0.722	0.185	0.648	0.167	0.148	0.222
Gnamptogenys sp.7	SP	1.148	0.37	0.315	0.685	0.241	0.648	0.204	0.148	0.185
Gnamptogenys sp.8	SP	1.8	0.486	0.429	1.314	0.4	1.086	0.286	0.229	0.4
Gnamptogenys sp.9	SP	1.407	0.426	0.37	0.759	0.167	0.852	0.315	0.074	0.426
Gnamptogenys striatula	MEP	1.478	0.389	0.404	1.207	0.256	0.789	0.322	0.167	0.437
Heteroponera dentinodis	MEP	1.194	0.343	0.434	0.68	0.234	0.68	0.257	0.171	0.383
Heteroponera dolo	MEP	1.646	0.469	0.577	0.96	0.32	0.943	0.36	0.217	0.531
Heteroponera inermis	MHP	1.194	0.337	0.377	0.754	0.223	0.669	0.229	0.183	0.36
Heteroponera mayri	MEP	1.103	0.297	0.383	0.634	0.217	0.64	0.223	0.16	0.343
Heteroponera microps	MHP	0.737	0.229	0.269	0.337	0.109	0.486	0.103	0.029	0.28
Heteroponera robusta	MEP	1.829	0.486	0.571	1.086	0.314	1	0.4	0.229	0.571
Hylomyrma balzani	MEP	1.089	0.47	0.244	0.774	0.233	0.77	0.233	0.219	0.096
Hylomyrma immanis	MEP	1.478	0.696	0.23	1.152	0.278	1.022	0.285	0.233	0.163
Hylomyrma reitteri	MEP	1.196	0.511	0.215	0.867	0.259	0.867	0.256	0.219	0.126

Hylomyrma sp.1	MEP	1.015	0.47	0.185	0.841	0.215	0.641	0.215	0.196	0.107
Hylomyrma sp.2	MEP	1.012	0.426	0.253	0.747	0.191	0.753	0.228	0.21	0.105
Hypoponera distinguenda	MEP	1.371	0.354	0.557	0.874	0.257	0.743	0.206	0.043	0.183
Hypoponera sp.1	MEP	1.526	0.343	0.56	1.091	0.257	0.76	0.217	0.086	0.229
Hypoponera sp.1 A	MEP	1.486	0.329	0.529	1.029	0.257	0.729	0.229	0.086	0.229
Hypoponera sp.10	SHP	0.846	0.185	0.327	0.63	0.154	0.451	0.123	0.037	0.13
Hypoponera sp.11	SHP	1.022	0.281	0.363	0.563	0.174	0.526	0.152	0.033	0.119
Hypoponera sp.12	SHP	1.196	0.3	0.448	0.796	0.207	0.626	0.174	0.033	0.178
Hypoponera sp.13	SHP	0.837	0.207	0.293	0.5	0.13	0.43	0.115	0.019	0.126
Hypoponera sp.14	SHP	0.841	0.215	0.296	0.485	0.141	0.459	0.119	0.02	0.104
Hypoponera sp.15	SHP	0.904	0.215	0.33	0.552	0.156	0.5	0.126	0.019	0.122
Hypoponera sp.16	SHP	0.796	0.196	0.226	0.478	0.152	0.444	0.111	0.02	0.115
Hypoponera sp.16 A	SHP	0.73	0.185	0.264	0.378	0.13	0.385	0.093	0.019	0.078
Hypoponera sp.17	SHP	1.033	0.263	0.37	0.57	0.193	0.567	0.144	0.037	0.119
Hypoponera sp.18	SHP	0.974	0.233	0.374	0.63	0.17	0.544	0.156	0.023	0.137
Hypoponera sp.19	SHP	0.904	0.193	0.337	0.667	0.182	0.482	0.141	0.059	0.144
Hypoponera sp.2	SHP	1.103	0.263	0.394	0.669	0.2	0.589	0.126	0.047	0.143
Hypoponera sp.20	SHP	1.008	0.235	0.394	0.638	0.196	0.553	0.161	0.033	0.151
Hypoponera sp.21	SHP	0.951	0.216	0.352	0.58	0.182	0.531	0.133	0.02	0.136
Hypoponera sp.21 A	SHP	0.929	0.219	0.355	0.62	0.185	0.525	0.139	0.022	0.13
Hypoponera sp.22	SHP	0.799	0.19	0.28	0.481	0.16	0.433	0.118	0.019	0.116
Hypoponera sp.23	SHP	0.622	0.148	0.23	0.322	0.115	0.348	0.085	0.005	0.078
Hypoponera sp.24	SHP	0.604	0.167	0.193	0.311	0.119	0.344	0.093	0.017	0.063
Hypoponera sp.25	SHP	0.66	0.16	0.227	0.352	0.118	0.35	0.097	0.019	0.081
Hypoponera sp.26	SHP	0.735	0.161	0.233	0.458	0.132	0.392	0.119	0.019	0.108
Hypoponera sp.27	SHP	0.529	0.132	0.183	0.291	0.108	0.288	0.074	0.017	0.042
Hypoponera sp.28	SHP	0.719	0.193	0.252	0.426	0.163	0.411	0.107	0.019	0.067
Hypoponera sp.29	SHP	0.982	0.296	0.296	0.519	0.167	0.482	0.13	0.037	0.093
Hypoponera sp.3	LEP	1.85	0.471	0.664	1.143	0.329	1.021	0.25	0.079	0.286
Hypoponera sp.30	SHP	0.519	0.13	0.167	0.278	0.093	0.278	0.093	0.015	0.037
Hypoponera sp.31	SHP	1.019	0.259	0.37	0.648	0.204	0.556	0.148	0.019	0.13
Hypoponera sp.4	SHP	1.149	0.286	0.411	0.703	0.211	0.617	0.137	0.057	0.154
Hypoponera sp.4 A	SHP	1.097	0.28	0.406	0.686	0.189	0.554	0.137	0.042	0.137
Hypoponera sp.5	SHP	1.229	0.343	0.514	0.8	0.229	0.629	0.143	0.029	0.2
Hypoponera sp.5 A	SHP	1.023	0.269	0.4	0.674	0.183	0.537	0.137	0.029	0.137
Hypoponera sp.5 B	SHP	0.981	0.238	0.39	0.648	0.181	0.543	0.143	0.029	0.133
Hypoponera sp.6	SHP	1.203	0.305	0.448	0.756	0.225	0.625	0.162	0.06	0.162
Hypoponera sp.6 A	SHP	1.029	0.257	0.386	0.657	0.171	0.529	0.143	0.079	0.143
Hypoponera sp.6 B	SHP	1.043	0.271	0.371	0.657	0.171	0.514	0.129	0.071	0.143

Hypoponera sp.7	SHP	1.771	0.429	0.6	1.114	0.314	0.943	0.257	0.057	0.257
Hypoponera sp.8	SHP	1	0.257	0.371	0.686	0.171	0.571	0.143	0.029	0.143
Hypoponera sp.9	SHP	1.269	0.36	0.497	0.731	0.223	0.674	0.16	0.029	0.149
Labidus coecus	N	1.53	0.367	0.313	1.302	0.359	0.838	0.132	0.025	0.517
Labidus praedatur	N	1.407	0.324	0.241	1.407	0.306	0.824	0.194	0.056	0.37
Lachnomyrmex nordestinus	MHP	0.715	0.259	0.152	0.519	0.174	0.541	0.167	0.1	0.074
Lachnomyrmex plaumanni	MHP	0.719	0.252	0.148	0.493	0.185	0.522	0.17	0.107	0.074
Lachnomyrmex victori	MHP	0.848	0.296	0.17	0.604	0.207	0.596	0.182	0.137	0.074
Leptogenys crudelis	SP	1.771	0.6	0.571	1.286	0.143	0.571	0.314	0.229	0.314
Leptogenys sp.1	SP	1	0.429	0.314	0.629	0.086	0.457	0.171	0.057	0.143
Leptogenys sp.2	SP	1.2	0.429	0.4	0.843	0.086	0.471	0.229	0.143	0.129
Leptogenys sp.3	SP	1.343	0.486	0.429	0.857	0.114	0.543	0.257	0.086	0.171
Leptogenys sp.4	SP	1.257	0.467	0.381	0.81	0.114	0.476	0.229	0.114	0.152
Leptogenys unistimulosa	SP	3.051	0.811	1.007	2.286	0.149	0.943	0.303	0.434	0.291
Leptothorax sp.1	G	0.889	0.315	0.204	0.574	0.204	0.63	0.185	0.167	0.167
Leptothorax sp.2	G	0.741	0.315	0.167	0.5	0.167	0.5	0.167	0.13	0.148
Leptothorax sp.3	G	0.982	0.37	0.241	0.556	0.204	0.63	0.222	0.148	0.185
Linepithema aff. *flavescens*	G	0.667	0.13	0.148	0.482	0.111	0.296	0.13	0.093	0.093
Linepithema aff. *micans*	G	0.843	0.139	0.176	0.685	0.167	0.324	0.16	0.127	0.13
Linepithema iniquum	G	0.901	0.167	0.176	0.722	0.167	0.34	0.161	0.148	0.142
Linepithema pullex	G	0.626	0.13	0.156	0.43	0.115	0.278	0.13	0.096	0.085
Linepithema sp.2	G	0.656	0.124	0.14	0.458	0.122	0.275	0.122	0.095	0.074
Linepithema sp.3	G	0.793	0.148	0.173	0.639	0.145	0.312	0.148	0.111	0.099
Linepithema sp.5	G	0.667	0.185	0.167	0.611	0.13	0.278	0.13	0.093	0.093
Linepithema sp.8	G	0.75	0.154	0.167	0.556	0.139	0.315	0.145	0.123	0.105
Megalomyrmex drifti	MEP	0.809	0.315	0.247	0.627	0.139	0.429	0.133	0.154	0.114
Megalomyrmex goeldii	MEP	1.022	0.4	0.285	0.867	0.174	0.582	0.156	0.122	0.196
Megalomyrmex iheringi	MEP	1.256	0.437	0.319	1.178	0.204	0.663	0.207	0.189	0.248
Megalomyrmex incisus	MEP	0.957	0.401	0.284	0.519	0.136	0.475	0.123	0.216	0.389
Megalomyrmex myops	MEP	0.769	0.343	0.241	0.611	0.176	0.5	0.093	0.074	0.167
Megalomyrmex pusillus	MEP	1.156	0.444	0.333	1.022	0.204	0.67	0.181	0.163	0.193
Megalomyrmex silvestrii	MEP	1.2	0.511	0.33	1.089	0.17	0.474	0.156	0.233	0.17
Monomorium floricola	SHG	0.426	0.167	0.111	0.241	0.056	0.278	0.111	0.028	0.093
Mycetarotes carinatus	FG	1.056	0.185	0.185	0.944	0.167	0.648	0.167	0.111	0.278
Mycocepurus pr. *smith*	FG	0.793	0.185	0.137	0.626	0.163	0.537	0.144	0.119	0.241
Mycocepurus sp.1	FG	1.074	0.204	0.185	0.982	0.185	0.648	0.241	0.167	0.278
Myrmelachista sp.1	G	0.759	0.213	0.204	0.546	0.13	0.491	0.148	0.139	0.222
Myrmelachista sp.2	G	0.556	0.148	0.148	0.352	0.093	0.352	0.111	0.074	0.111
Myrmicocrypta sp.1	FG	1.019	0.352	0.19	0.884	0.19	0.648	0.218	0.097	0.296

Myrmicocrypta sp.2	FG	1.056	0.356	0.189	0.9	0.211	0.682	0.215	0.074	0.293
Myrmicocrypta sp.3	FG	0.856	0.255	0.167	0.819	0.162	0.593	0.204	0.074	0.282
Myrmicocrypta sp.4	FG	0.852	0.269	0.148	0.722	0.185	0.574	0.185	0.056	0.278
Myrmicocrypta sp.5	FG	1.033	0.311	0.174	0.937	0.189	0.711	0.244	0.059	0.326
Neivamyrmex punctaticeps	N	0.852	0.226	0.211	0.541	0.189	0.504	0.048	0	0.315
Neivamyrmex sp.1	N	0.648	0.185	0.167	0.352	0.111	0.389	0.037	0	0.259
Neivamyrmex sp.2	N	0.938	0.228	0.198	0.747	0.204	0.537	0.074	0.015	0.327
Neivamyrmex sp.3	N	0.556	0.167	0.13	0.296	0.093	NA	0.037	0.001	NA
Ochetomyrmex subpolitus	G	0.731	0.264	0.213	0.569	0.148	0.5	0.181	0.153	0.116
Octostruma balzani	DP	0.611	0.216	0.173	0.377	0.093	0.494	0.185	0.049	0.185
Octostruma iheringi	DP	0.781	0.326	0.215	0.552	0.1	0.552	0.226	0.035	0.256
Octostruma petiolata	DP	0.863	0.337	0.233	0.559	0.122	0.678	0.222	0.046	0.259
Octostruma rugifera	DP	0.568	0.213	0.164	0.383	0.069	0.478	0.164	0.049	0.188
Octostruma simoni	DP	0.641	0.267	0.17	0.389	0.082	0.467	0.178	0.022	0.211
Octostruma sp.2	DP	0.77	0.322	0.226	0.493	0.1	0.511	0.226	0.037	0.263
Octostruma sp.3	DP	0.748	0.296	0.207	0.485	0.1	0.533	0.215	0.035	0.252
Octostruma sp.5	DP	0.763	0.322	0.23	0.507	0.104	0.544	0.207	0.037	0.245
Octostruma sp.7	DP	0.759	0.306	0.222	0.482	0.12	0.546	0.185	0.032	0.296
Octostruma stenognatha	DP	0.595	0.249	0.153	0.384	0.082	0.384	0.188	0.057	0.204
Odontomachus affinis	LEP	3.476	0.638	0.829	3.152	0.4	1.581	0.419	0.429	0.724
Odontomachus brunneus	LEP	3.229	0.61	0.819	2.838	0.438	1.562	0.381	0.467	0.762
Odontomachus chelifer	LEP	4.276	0.81	0.876	4.076	0.514	1.829	0.448	0.543	0.857
Odontomachus meinerti	LEP	2.531	0.469	0.646	2.086	0.314	1.034	0.394	0.32	0.566
Oxyepoecus browni	G	0.615	0.233	0.233	0.382	0.07	0.415	0.137	0.074	0.093
Oxyepoecus bruchi	G	0.611	0.241	0.185	0.389	0.074	0.389	0.167	0.074	0.111
Oxyepoecus crassinodus	G	0.659	0.241	0.207	0.396	0.1	0.452	0.148	0.074	0.115
Oxyepoecus longicephalus	G	0.593	0.241	0.167	0.315	0.093	0.37	0.111	0.074	0.093
Oxyepoecus myops	G	0.563	0.208	0.167	0.347	0.093	0.38	0.104	0.058	0.1
Oxyepoecus plaumanni	G	0.578	0.226	0.178	0.356	0.1	0.396	0.137	0.074	0.096
Oxyepoecus punctifrons	G	0.719	0.274	0.215	0.5	0.104	0.448	0.152	0.1	0.133
Oxyepoecus rastratus	G	0.559	0.207	0.164	0.346	0.093	0.383	0.111	0.065	0.096
Oxyepoecus reticulatus	G	0.599	0.222	0.179	0.383	0.099	0.407	0.142	0.062	0.105
Oxyepoecus rosai	G	0.571	0.222	0.173	0.364	0.099	0.386	0.102	0.068	0.102
Oxyepoecus vezenyii	G	0.593	0.222	0.185	0.37	0.111	0.407	0.148	0.074	0.111
Pachycondyla arhuaca	MEP	1.766	0.423	0.714	1.149	0.314	0.971	0.269	0.137	0.149
Pachycondyla bucki	LEP	2.114	0.486	0.846	1.406	0.326	0.931	0.349	0.229	0.246
Pachycondyla constricta	LEP	2.491	0.589	0.926	1.869	0.36	1	0.349	0.371	0.257
Pachycondyla ferruginea	MHP	1.686	0.48	0.691	0.931	0.354	1	0.177	0.12	0.166
Pachycondyla gilberti	MEP	1.486	0.364	0.5	0.857	0.279	0.871	0.157	0.057	0.114

Pachycondyla harpax	LEP	2.686	0.691	1.046	1.646	0.514	1.451	0.349	0.28	0.251
Pachycondyla lenis	LEP	2.874	0.8	1.194	1.863	0.571	1.577	0.4	0.291	0.326
Pachycondyla lunaris	MEP	1.714	0.514	0.714	1.029	0.343	1.057	0.171	0.143	0.171
Pachycondyla metanotalis	LEP	3.257	0.886	1.286	2.2	0.629	1.814	0.486	0.486	0.314
Pachycondyla pr. *stigma*	MHP	1.349	0.343	0.486	0.8	0.24	0.806	0.206	0.069	0.12
Pachycondyla pr. *venusta*	MEP	2.029	0.533	0.724	1.733	0.391	1.038	0.295	0.333	0.21
Pachycondyla sp.1	MEP	2.4	0.543	0.971	1.886	0.343	1	0.257	0.257	0.257
Pachycondyla sp.2	MEP	1.371	0.343	0.514	0.8	0.257	0.771	0.257	0.086	0.143
Pachycondyla sp.3	MEP	1.286	0.314	0.486	0.743	0.229	0.743	0.229	0.057	0.114
Pachycondyla sp.4	MEP	1.429	0.343	0.514	0.857	0.286	0.857	0.257	0.057	0.143
Pachycondyla sp.5	MEP	0.778	0.185	0.241	0.37	0.111	0.407	0.093	0.015	0.074
Pachycondyla striata	LEP	4.267	1.114	1.629	3.162	0.762	2.19	0.543	0.514	0.362
Pachycondyla unidentata	LEP	2.343	0.743	0.714	1.743	0.486	1.143	0.657	0.371	0.371
Pachycondyla venusta	MEP	1.674	0.463	0.64	1.286	0.28	0.846	0.257	0.286	0.154
Paratrechina longicornis	G	0.907	0.157	0.167	1.037	0.093	0.296	0.157	0.176	0.176
Nylanderia sp.1	G	0.767	0.189	0.178	0.707	0.1	0.33	0.156	0.126	0.148
Nylanderia sp.2	G	0.678	0.163	0.148	0.604	0.085	0.3	0.141	0.104	0.126
Nylanderia sp.3	G	0.772	0.167	0.174	0.719	0.093	0.33	0.154	0.12	0.13
Nylanderia sp.4	G	0.917	0.185	0.222	0.917	0.148	0.37	0.204	0.204	0.185
Phalacromyrmex fugax	DP	0.963	0.452	0.274	0.607	0.215	0.97	0.296	0.082	0.37
Pheidole sp.1	G	0.492	0.159	0.111	0.376	0.114	0.381	0.124	0.083	0.074
Pheidole sp.10	G	0.407	0.116	0.079	0.329	0.093	0.329	0.111	0.074	0.051
Pheidole sp.11	G	0.552	0.174	0.107	0.415	0.13	0.422	0.141	0.074	0.111
Pheidole sp.12	G	0.537	0.163	0.104	0.478	0.115	0.4	0.133	0.078	0.085
Pheidole sp.13	G	0.735	0.253	0.142	0.648	0.191	0.488	0.185	0.117	0.154
Pheidole sp.14	G	0.544	0.159	0.093	0.552	0.107	0.389	0.133	0.089	0.085
Pheidole sp.15	G	0.593	0.148	0.111	0.389	0.167	0.5	0.204	0.093	0.111
Pheidole sp.16	G	0.582	0.185	0.107	0.43	0.122	0.441	0.137	0.082	0.115
Pheidole sp.17	G	0.611	0.167	0.107	0.47	0.144	0.47	0.148	0.082	0.1
Pheidole sp.18	G	0.704	0.2	0.126	0.522	0.178	0.537	0.144	0.085	0.115
Pheidole sp.19	G	0.83	0.215	0.133	0.63	0.193	0.574	0.17	0.1	0.144
Pheidole sp.2	G	0.444	0.159	0.1	0.333	0.111	0.367	0.111	0.063	0.059
Pheidole sp.20	G	0.756	0.2	0.126	0.6	0.182	0.526	0.144	0.087	0.141
Pheidole sp.21	G	0.806	0.25	0.185	0.546	0.315	0.852	0.241	0.093	0.176
Pheidole sp.22	G	0.815	0.207	0.148	0.763	0.185	0.544	0.207	0.163	0.174
Pheidole sp.23	G	0.756	0.204	0.122	0.733	0.156	0.504	0.174	0.144	0.178
Pheidole sp.24	G	0.726	0.196	0.126	0.681	0.137	0.456	0.159	0.122	0.152
Pheidole sp.25	G	0.689	0.178	0.107	0.711	0.126	0.396	0.148	0.137	0.137
Pheidole sp.26	G	0.963	0.259	0.148	0.852	0.222	0.648	0.185	0.111	0.167

Pheidole sp.27	G	0.534	0.148	0.095	0.429	0.135	0.426	0.127	0.077	0.085
Pheidole sp.28	G	0.769	0.194	0.117	0.633	0.185	0.531	0.157	0.086	0.133
Pheidole sp.29	G	0.567	0.148	0.093	0.467	0.115	0.4	0.133	0.093	0.107
Pheidole sp.3	G	0.496	0.163	0.119	0.415	0.107	0.396	0.137	0.093	0.082
Pheidole sp.30	MEP	1.174	0.289	0.167	1.019	0.274	0.741	0.2	0.152	0.182
Pheidole sp.31	MEP	1.144	0.296	0.174	1.033	0.311	0.793	0.2	0.126	0.207
Pheidole sp.32	MEP	1.194	0.333	0.185	1.102	0.333	0.843	0.204	0.12	0.213
Pheidole sp.33	G	0.778	0.222	0.13	0.611	0.185	0.556	0.148	0.111	0.148
Pheidole sp.34	G	0.537	0.163	0.093	0.415	0.133	0.433	0.111	0.054	0.1
Pheidole sp.35	G	0.53	0.159	0.093	0.407	0.137	0.426	0.119	0.048	0.104
Pheidole sp.36	G	0.474	0.144	0.078	0.363	0.115	0.378	0.115	0.048	0.089
Pheidole sp.37	G	0.989	0.252	0.141	0.889	0.204	0.582	0.174	0.126	0.163
Pheidole sp.38	G	0.837	0.207	0.119	0.737	0.189	0.53	0.144	0.104	0.141
Pheidole sp.39	G	0.796	0.2	0.115	0.689	0.178	0.507	0.126	0.1	0.13
Pheidole sp.4	G	0.47	0.159	0.115	0.4	0.1	0.382	0.13	0.082	0.085
Pheidole sp.40	MEP	1.088	0.255	0.153	1.028	0.213	0.611	0.204	0.125	0.181
Pheidole sp.41	MEP	1.093	0.296	0.191	1	0.247	0.809	0.198	0.074	0.241
Pheidole sp.42	G	0.917	0.231	0.153	0.833	0.208	0.648	0.157	0.06	0.208
Pheidole sp.43	G	0.837	0.226	0.144	0.789	0.167	0.526	0.185	0.137	0.17
Pheidole sp.44	G	0.852	0.235	0.136	0.867	0.154	0.503	0.179	0.139	0.173
Pheidole sp.45	MEP	1.074	0.278	0.148	1.222	0.185	0.482	0.222	0.167	0.185
Pheidole sp.46	G	0.737	0.196	0.126	0.73	0.144	0.489	0.152	0.111	0.156
Pheidole sp.47	MEP	1.023	0.269	0.143	1.213	0.148	0.472	0.185	0.157	0.167
Pheidole sp.48	G	0.781	0.213	0.136	0.79	0.154	0.469	0.179	0.139	0.161
Pheidole sp.49	G	0.807	0.185	0.126	0.752	0.174	0.537	0.163	0.096	0.141
Pheidole sp.5	G	0.5	0.148	0.102	0.417	0.102	0.389	0.13	0.074	0.083
Pheidole sp.50	G	0.741	0.185	0.148	0.741	0.148	0.5	0.185	0.13	0.167
Pheidole sp.51	G	0.744	0.207	0.122	0.756	0.141	0.367	0.159	0.119	0.152
Pheidole sp.52	MEP	1.185	0.278	0.167	1.148	0.185	0.593	0.185	0.185	0.222
Pheidole sp.53	G	0.904	0.293	0.196	0.944	0.207	0.644	0.215	0.148	0.189
Pheidole sp.54	G	0.874	0.244	0.133	0.926	0.174	0.526	0.181	0.148	0.185
Pheidole sp.55	G	0.738	0.207	0.117	0.753	0.154	0.457	0.164	0.12	0.157
Pheidole sp.56	MEP	1.148	0.306	0.157	1.287	0.185	0.546	0.204	0.167	0.204
Pheidole sp.57	MEP	1.102	0.272	0.145	1.228	0.179	0.525	0.182	0.157	0.204
Pheidole sp.58	MEP	1.074	0.287	0.148	1.25	0.185	0.482	0.194	0.167	0.185
Pheidole sp.59	G	0.741	0.222	0.13	0.759	0.148	0.463	0.148	0.13	0.148
Pheidole sp.6	G	0.393	0.133	0.1	0.322	0.089	0.33	0.111	0.07	0.048
Pheidole sp.60	G	0.676	0.185	0.111	0.713	0.125	0.407	0.148	0.116	0.116
Pheidole sp.61	G	0.954	0.231	0.134	0.972	0.194	0.565	0.194	0.125	0.171

Pheidole sp.62	G	0.852	0.241	0.148	0.833	0.185	0.611	0.185	0.167	0.204
Pheidole sp.63	MEP	1.463	0.358	0.191	1.482	0.278	0.877	0.278	0.161	0.265
Pheidole sp.64	G	0.578	0.163	0.104	0.433	0.122	0.433	0.137	0.089	0.119
Pheidole sp.65	MEP	1.241	0.296	0.189	1.052	0.285	0.804	0.244	0.152	0.219
Pheidole sp.66	MEP	1.111	0.241	0.185	0.926	0.222	0.722	0.204	0.13	0.111
Pheidole sp.67	G	0.741	0.215	0.119	0.581	0.174	0.53	0.148	0.037	0.133
Pheidole sp.68	G	0.907	0.282	0.185	1.245	0.255	0.769	0.245	0.143	0.213
Pheidole sp.69	G	0.9	0.359	0.185	0.844	0.207	0.7	0.196	0.126	0.163
Pheidole sp.7	G	0.421	0.134	0.083	0.352	0.088	0.347	0.111	0.069	0.069
Pheidole sp.70	G	0.87	0.241	0.167	0.574	0.315	0.87	0.204	0.13	0.167
Pheidole sp.71	SHG	0.917	0.398	0.278	0.713	0.167	0.556	0.241	0.093	0.176
Pheidole sp.8	G	0.359	0.115	0.078	0.278	0.082	0.304	0.1	0.063	0.048
Pheidole sp.9	G	0.506	0.154	0.099	0.401	0.111	0.395	0.123	0.08	0.08
Prionopelta antillana	SP	0.62	0.151	0.194	0.312	0.056	0.417	0.117	0.022	0.231
Prionopelta punctulata	SP	0.602	0.161	0.182	0.278	0.054	0.395	0.108	0.013	0.216
Prionopelta sp.1	SP	0.741	0.199	0.218	0.338	0.056	0.482	0.116	0.025	0.25
Prionopelta sp.2	SP	0.463	0.167	0.167	0.185	0.037	0.278	0.037	0.009	0.222
Proceratium brasiliense	SP	1.033	0.348	0.322	0.711	0.23	0.719	0.193	0.035	0.259
Procryptocerus convergens	V	1.685	0.398	0.343	1.028	0.213	1.111	0.278	0.333	0.306
Procryptocerus regularis	V	1.482	0.37	0.352	0.815	0.213	0.963	0.241	0.296	0.204
Pseudomyrmex sp.1	MEP	1.519	0.528	0.269	0.769	0.269	0.583	0.185	0.491	0.157
Pseudomyrmex sp.2	MEP	1.691	0.698	0.414	1.068	0.327	0.679	0.154	0.741	0.154
Pseudomyrmex sp.3	MEP	1.852	0.778	0.296	1.056	0.315	0.685	0.167	0.583	0.222
Pseudomyrmex sp.4	MEP	1.713	0.676	0.361	0.982	0.306	0.722	0.157	0.639	0.213
Pyramica appretiata	DP	0.526	0.207	0.13	0.341	0.082	0.459	0.189	0.046	0.219
Pyramica cf. *teratrix*	DP	0.415	0.159	0.096	0.259	0.059	0.396	0.141	0.031	0.182
Pyramica cf. *urrhobia*	DP	0.472	0.148	0.102	0.315	0.037	0.278	0.148	0.037	0.176
Pyramica cf. *villiersi*	DP	0.5	0.204	0.093	0.315	0.037	0.278	0.13	0.019	0.185
Pyramica crassicornis	DP	0.54	0.201	0.13	0.38	0.032	0.377	0.16	0.039	0.204
Pyramica denticulata	DP	0.444	0.143	0.09	0.283	0.029	0.275	0.127	0.037	0.177
Pyramica dentinasis	DP	0.79	0.309	0.173	0.506	0.056	0.574	0.253	0.062	0.284
Pyramica pr. *lygatrix*	DP	0.565	0.259	0.13	0.38	0.042	0.352	0.167	0.042	0.204
Pyramica reticeps	DP	0.528	0.222	0.12	0.333	0.065	0.278	0.167	0.019	0.194
Pyramica rugithorax	DP	0.688	0.299	0.136	0.466	0.04	0.432	0.213	0.037	0.318
Pyramica schulzi	DP	0.491	0.157	0.102	0.306	0.019	0.269	0.167	0.056	0.167
Pyramica sp. nov. C	DP	0.493	0.174	0.111	0.27	0.046	0.385	0.182	0.019	0.182
Pyramica sp.1	DP	0.482	0.204	0.111	0.296	0.056	0.444	0.167	0.037	0.222
Pyramica sp.11	DP	0.463	0.204	0.093	0.296	0.037	0.259	0.148	0.037	0.185
Pyramica sp.12	DP	0.426	0.148	0.093	0.259	0.056	0.259	0.148	0.009	0.185

Pyramica sp.13	DP	0.472	0.157	0.111	0.296	0.019	0.269	0.167	0.005	0.222
Pyramica sp.14	DP	0.474	0.17	0.096	0.304	0.044	0.278	0.152	0.037	0.185
Pyramica sp.15	DP	0.472	0.167	0.13	0.343	0.023	0.269	0.148	0.037	0.185
Pyramica sp.16	DP	0.444	0.167	0.111	0.278	0.019	0.222	0.148	0.037	0.167
Pyramica sp.17	DP	0.519	0.204	0.111	0.352	0.037	0.315	0.167	0.019	0.204
Pyramica sp.18	DP	0.419	0.159	0.1	0.274	0.037	0.296	0.104	0.03	0.189
Pyramica sp.4	DP	0.459	0.167	0.133	0.304	0.031	0.311	0.167	0.022	0.207
Pyramica sp.6	DP	0.522	0.219	0.093	0.341	0.056	0.304	0.148	0.035	0.189
Pyramica sp.7	DP	0.352	0.148	0.093	0.185	0.019	0.185	0.111	0.019	0.185
Pyramica sp.8	DP	0.5	0.167	0.111	0.302	0.037	0.272	0.13	0.053	0.167
Pyramica sp.9	DP	0.444	0.179	0.111	0.327	0.053	0.29	0.148	0.037	0.185
Pyramica splendens	DP	0.667	0.3	0.133	0.456	0.052	0.315	0.204	0.037	0.248
Pyramica subdentata	DP	0.5	0.2	0.111	0.37	0.052	0.322	0.137	0.037	0.189
Pyramica tanymastax	DP	0.438	0.17	0.105	0.29	0.028	0.265	0.127	0.019	0.185
Rhopalothrix bruchi	DP	0.496	0.137	0.159	0.263	0.078	0.411	0.137	0.017	0.211
Rhopalothrix rosae	DP	0.407	0.111	0.111	0.204	0.056	0.361	0.111	0.006	0.176
Rhopalothrix sp.G	DP	0.449	0.144	0.139	0.259	0.069	0.444	0.139	0.009	0.19
Rhopalothrix sp.L	DP	0.463	0.167	0.167	0.278	0.074	0.407	0.148	0.019	0.241
Rogeria preminula	MHP	0.565	0.222	0.148	0.398	0.13	0.463	0.139	0.037	0.13
Rogeria sp.	G	0.444	0.167	0.13	0.296	0.111	0.389	0.074	0.019	0.111
Rogeria sp.1	MHP	0.611	0.244	0.163	0.415	0.115	0.437	0.163	0.056	0.107
Rogeria sp.10	MHP	0.574	0.241	0.148	0.426	0.111	0.407	0.148	0.056	0.13
Rogeria sp.11	MHP	0.407	0.148	0.111	0.241	0.093	0.278	0.111	0.037	0.074
Rogeria sp.12	MHP	0.611	0.274	0.207	0.437	0.115	0.441	0.152	0.07	0.167
Rogeria sp.2	MHP	0.693	0.289	0.207	0.456	0.163	0.552	0.182	0.059	0.174
Rogeria sp.3	MHP	0.867	0.393	0.233	0.663	0.178	0.641	0.189	0.07	0.185
Rogeria sp.4	MHP	0.833	0.37	0.222	0.585	0.17	0.611	0.193	0.048	0.185
Rogeria sp.5	MHP	0.458	0.148	0.125	0.296	0.097	0.37	0.139	0.019	0.111
Rogeria sp.6	MHP	1.065	0.491	0.269	0.778	0.241	0.759	0.241	0.102	0.213
Rogeria sp.7	MHP	0.459	0.182	0.111	0.3	0.093	0.33	0.115	0.037	0.088
Rogeria sp.8	MHP	0.458	0.213	0.13	0.319	0.088	0.347	0.102	0.039	0.093
Rogeria sp.9	MHP	0.556	0.241	0.148	0.426	0.102	0.398	0.12	0.037	0.139
Rogeria subarmata	MHP	0.898	0.375	0.204	0.63	0.171	0.648	0.213	0.143	0.171
Sericomyrmex sp.1	FG	1.296	0.304	0.237	1.107	0.248	0.878	0.259	0.159	0.089
Sericomyrmex sp.2	FG	1.194	0.259	0.208	0.958	0.218	0.769	0.241	0.148	0.074
Sericomyrmex sp.3	FG	1.417	0.292	0.25	1.148	0.264	0.894	0.292	0.157	0.12
Sericomyrmex sp.4	FG	1.097	0.218	0.19	0.833	0.208	0.699	0.235	0.148	0.111
Solenopsis pr. *invicta*	MEP	1.093	0.359	0.285	0.915	0.219	0.73	0.274	0.148	0.222
Solenopsis pr. *terricola*	SHG	0.393	0.137	0.133	0.2	0.082	0.285	0.115	0.037	0.074

Solenopsis pr. *terricola* A	SHG	0.382	0.133	0.133	0.204	0.074	0.282	0.104	0.037	0.074
Solenopsis pr. *terricola* B	SHG	0.385	0.137	0.122	0.196	0.082	0.282	0.111	0.037	0.067
Solenopsis pr. *terricola* C	SHG	0.364	0.117	0.111	0.167	0.062	0.265	0.099	0.034	0.056
Solenopsis pr. *terricola* D	SHG	0.389	0.111	0.111	0.204	0.074	0.259	0.093	0.037	0.074
Solenopsis sp.1	SHG	0.43	0.137	0.119	0.267	0.063	0.296	0.107	0.028	0.078
Solenopsis sp.10	MEP	1.181	0.415	0.304	1.015	0.219	0.759	0.285	0.13	0.248
Solenopsis sp.13	SHG	0.526	0.193	0.152	0.344	0.074	0.337	0.13	0.037	0.078
Solenopsis sp.14	SHG	0.63	0.241	0.204	0.407	0.093	0.444	0.148	0.056	0.13
Solenopsis sp.15	SHG	0.537	0.204	0.176	0.324	0.093	0.398	0.148	0.037	0.102
Solenopsis sp.16	SHG	0.759	0.259	0.185	0.463	0.148	0.556	0.222	0.074	0.13
Solenopsis sp.17	SHG	0.63	0.222	0.167	0.407	0.13	0.444	0.167	0.056	0.111
Solenopsis sp.18	SHG	0.568	0.204	0.167	0.42	0.074	0.407	0.161	0.037	0.099
Solenopsis sp.19	SHG	0.556	0.204	0.167	0.352	0.093	0.389	0.13	0.046	0.083
Solenopsis sp.2	SHG	0.478	0.17	0.148	0.278	0.078	0.352	0.13	0.027	0.089
Solenopsis sp.20	SHG	0.482	0.167	0.167	0.278	0.093	0.352	0.13	0.037	0.074
Solenopsis sp.21	SHG	0.519	0.204	0.167	0.389	0.111	0.37	0.148	0.037	0.093
Solenopsis sp.22	SHG	0.444	0.167	0.148	0.259	0.093	0.333	0.13	0.037	0.074
Solenopsis sp.23	SHG	0.546	0.213	0.139	0.352	0.093	0.389	0.139	0.037	0.102
Solenopsis sp.24	SHG	0.544	0.196	0.163	0.37	0.089	0.385	0.141	0.044	0.093
Solenopsis sp.25	SHG	0.34	0.117	0.096	0.191	0.049	0.238	0.102	0.02	0.056
Solenopsis sp.26	SHG	0.389	0.12	0.093	0.194	0.056	0.259	0.083	0.037	0.056
Solenopsis sp.27	SHG	0.426	0.148	0.111	0.278	0.074	0.296	0.111	0.028	0.074
Solenopsis sp.28	SHG	0.444	0.167	0.148	0.241	0.074	0.333	0.111	0.037	0.074
Solenopsis sp.29	SHG	0.407	0.148	0.13	0.222	0.074	0.302	0.123	0.037	0.093
Solenopsis sp.2A	SHG	0.407	0.139	0.12	0.213	0.065	0.296	0.13	0.019	0.074
Solenopsis sp.2B	SHG	0.537	0.194	0.176	0.306	0.093	0.398	0.148	0.037	0.083
Solenopsis sp.3	SHG	0.352	0.126	0.107	0.185	0.056	0.274	0.096	0.019	0.056
Solenopsis sp.3 A	SHG	0.338	0.106	0.097	0.181	0.051	0.245	0.088	0.019	0.056
Solenopsis sp.3 B	SHG	0.367	0.111	0.107	0.204	0.052	0.289	0.1	0.03	0.059
Solenopsis sp.3 C	SHG	0.356	0.126	0.122	0.193	0.059	0.27	0.1	0.037	0.067
Solenopsis sp.30	SHG	0.463	0.17	0.115	0.3	0.074	0.315	0.122	0.037	0.085
Solenopsis sp.31	SHG	0.4	0.148	0.111	0.267	0.067	0.282	0.104	0.037	0.07
Solenopsis sp.32	SHG	0.463	0.204	0.148	0.278	0.074	0.333	0.13	0.028	0.074
Solenopsis sp.33	SHG	0.377	0.123	0.111	0.235	0.056	0.272	0.105	0.025	0.062
Solenopsis sp.34	SHG	0.417	0.139	0.102	0.25	0.074	0.287	0.12	0.028	0.083
Solenopsis sp.35	SHG	0.407	0.139	0.12	0.232	0.083	0.296	0.102	0.037	0.065
Solenopsis sp.36	SHG	0.394	0.13	0.116	0.218	0.065	0.287	0.116	0.021	0.069
Solenopsis sp.37	SHG	0.454	0.157	0.13	0.306	0.065	0.315	0.111	0.037	0.093
Solenopsis sp.4	SHG	0.378	0.111	0.1	0.237	0.059	0.27	0.104	0.022	0.067

Solenopsis sp.5	SHG	0.411	0.137	0.13	0.222	0.067	0.304	0.111	0.031	0.07
Solenopsis sp.6	SHG	0.404	0.133	0.104	0.244	0.059	0.274	0.104	0.035	0.07
Solenopsis sp.7	SHG	0.463	0.163	0.122	0.296	0.07	0.311	0.122	0.034	0.085
Solenopsis sp.8	SHG	0.589	0.215	0.2	0.393	0.107	0.444	0.17	0.044	0.141
Solenopsis sp.9	SHG	0.526	0.196	0.152	0.378	0.089	0.381	0.141	0.034	0.104
Solenopsis subterranea	SHG	0.327	0.114	0.083	0.164	0.043	0.231	0.09	0.007	0.049
Solenopsis terricola	SHG	0.348	0.122	0.111	0.185	0.067	0.274	0.096	0.035	0.059
Sphinctomyrmex stali	SP	0.88	0.272	0.312	0.444	0.161	0.515	0.12	0.043	0.34
Stegomyrmex vizzotoi	SP	1.588	0.63	0.343	1.093	0.375	1.014	0.171	0.079	0.403
Stigmatomma armigerum	SP	1.509	0.586	0.509	0.799	0.164	0.917	0.105	0.042	0.528
Stigmatomma elongatum	SP	0.784	0.33	0.275	0.361	0.077	0.472	0.049	0.012	0.281
Stigmatomma sp.1	SP	1.593	0.593	0.537	0.815	0.167	0.944	0.111	0.074	0.556
Strumigenys cordovensis	DP	0.772	0.358	0.173	0.735	0.074	0.37	0.167	0.068	0.265
Strumigenys cosmostela	DP	0.63	0.244	0.13	0.456	0.059	0.337	0.13	0.035	0.237
Strumigenys elongata	DP	0.567	0.211	0.122	0.444	0.052	0.3	0.122	0.037	0.244
Strumigenys louisianae	DP	0.463	0.164	0.105	0.309	0.035	0.275	0.12	0.031	0.207
Strumigenys pr. *louisianae* A	DP	0.474	0.178	0.111	0.319	0.035	0.293	0.115	0.031	0.196
Strumigenys pr. *sanctipauli*	DP	0.969	0.37	0.167	0.883	0.086	0.395	0.198	0.068	0.278
Strumigenys pr. *schmalzi* B	DP	0.393	0.13	0.089	0.252	0.022	0.248	0.089	0.015	0.193
Strumigenys pr. *schmalzi* C	DP	0.37	0.148	0.086	0.247	0.022	0.247	0.105	0.043	0.173
Strumigenys precava	DP	0.917	0.38	0.157	0.833	0.093	0.454	0.167	0.056	0.324
Strumigenys saliens	DP	0.859	0.37	0.178	0.715	0.082	0.411	0.148	0.085	0.263
Strumigenys schmalzi	DP	0.404	0.148	0.089	0.263	0.03	0.248	0.096	0.011	0.2
Strumigenys smithi	DP	0.716	0.34	0.161	0.593	0.08	0.389	0.142	0.068	0.247
Strumigenys sp. nov. A	DP	0.819	0.374	0.156	0.715	0.067	0.385	0.148	0.082	0.282
Strumigenys sp. nov. F	DP	0.652	0.244	0.13	0.474	0.061	0.33	0.137	0.048	0.233
Strumigenys sp.10	DP	0.685	0.296	0.167	0.648	0.074	0.444	0.167	0.056	0.241
Strumigenys sp.11	DP	0.796	0.321	0.154	0.716	0.056	0.37	0.198	0.068	0.222
Strumigenys sp.12	DP	0.877	0.383	0.173	0.765	0.093	0.401	0.167	0.08	0.253
Strumigenys sp.13	DP	0.426	0.157	0.093	0.278	0.037	0.259	0.111	0.037	0.204
Strumigenys sp.14	DP	0.586	0.21	0.105	0.463	0.068	0.309	0.13	0.037	0.247
Strumigenys sp.15	DP	0.556	0.222	0.111	0.37	0.056	0.241	0.13	0.037	0.185
Strumigenys sp.16	DP	0.482	0.13	0.111	0.333	0.037	0.278	0.111	0.019	0.204
Strumigenys sp.17	DP	0.438	0.167	0.105	0.29	0.028	0.259	0.105	0.031	0.185
Strumigenys sp.18	DP	0.556	0.204	0.13	0.389	0.056	0.37	0.148	0.056	0.185
Strumigenys sp.19	DP	0.559	0.219	0.115	0.378	0.044	0.319	0.115	0.048	0.204
Strumigenys sp.2	DP	0.544	0.207	0.111	0.378	0.041	0.3	0.133	0.037	0.196
Strumigenys sp.3	DP	0.648	0.204	0.148	0.426	0.056	0.352	0.13	0.056	0.241
Strumigenys sp.4	DP	0.542	0.199	0.13	0.352	0.037	0.296	0.125	0.046	0.185

Strumigenys sp.5	DP	0.944	0.38	0.148	0.852	0.083	0.435	0.176	0.065	0.306
Strumigenys sp.7	DP	0.667	0.296	0.148	0.537	0.056	0.389	0.148	0.074	0.259
Strumigenys sp.8	DP	0.728	0.315	0.167	0.716	0.074	0.383	0.161	0.074	0.247
Strumigenys sp.9	DP	0.97	0.385	0.163	0.896	0.089	0.415	0.204	0.074	0.27
Tapinoma melanocephalum	G	0.491	0.1	0.096	0.441	0.139	0.278	0.114	0.105	0.09
Thaumatomyrmex atrox	SP	0.756	0.274	0.377	0.482	0.078	0.407	0.115	0.144	0.063
Thaumatomyrmex mutillatus	SP	1.237	0.415	0.574	0.915	0.17	0.811	0.219	0.263	0.119
Trachymyrmex kempfi	FG	1.185	0.259	0.167	1.019	0.185	0.667	0.241	0.148	0.167
Trachymyrmex sp.1	FG	1.278	0.287	0.204	1.157	0.194	0.722	0.241	0.148	0.139
Trachymyrmex sp.10	FG	1.5	0.315	0.204	1.5	0.222	0.796	0.259	0.167	0.222
Trachymyrmex sp.2	FG	1.611	0.356	0.233	1.5	0.278	0.919	0.333	0.196	0.248
Trachymyrmex sp.3	FG	1.366	0.292	0.194	1.259	0.222	0.787	0.269	0.181	0.19
Trachymyrmex sp.4	FG	1.37	0.278	0.204	1.241	0.241	0.852	0.296	0.148	0.167
Trachymyrmex sp.5	FG	2.043	0.352	0.29	1.957	0.287	1.086	0.352	0.259	0.235
Trachymyrmex sp.6	FG	1.093	0.204	0.148	0.944	0.185	0.63	0.222	0.148	0.185
Trachymyrmex sp.7	FG	0.996	0.182	0.156	0.852	0.17	0.593	0.215	0.122	0.141
Trachymyrmex sp.8	FG	1.611	0.315	0.222	1.519	0.241	0.926	0.315	0.204	0.222
Trachymyrmex sp.9	FG	1.194	0.241	0.157	1.009	0.194	0.694	0.278	0.139	0.139
Typhlomyrmex major	SP	0.827	0.247	0.201	0.438	0.176	0.552	0.164	0.017	0.201
Typhlomyrmex pusillus	SP	0.698	0.204	0.179	0.358	0.136	0.448	0.111	0.009	0.157
Typhlomyrmex rogenhoferi	SP	1.185	0.352	0.259	0.602	0.278	0.787	0.241	0.056	0.315
Typhlomyrmex sp.1	SP	0.463	0.148	0.13	0.222	0.074	0.278	0.074	0.002	0.13
Typhlomyrmex sp.2	SP	0.546	0.167	0.148	0.259	0.093	0.315	0.093	0.002	0.12
Wasmannia affinis	G	0.568	0.228	0.182	0.448	0.108	0.42	0.148	0.102	0.083
Wasmannia auropunctata	G	0.448	0.174	0.148	0.374	0.085	0.326	0.137	0.082	0.074
Wasmannia lutzi	G	0.522	0.207	0.167	0.381	0.1	0.348	0.156	0.104	0.067
Wasmannia rochai	G	0.444	0.2	0.156	0.352	0.085	0.33	0.104	0.085	0.07
Wasmannia scrobifera	G	0.5	0.215	0.167	0.378	0.107	0.389	0.152	0.115	0.085
Wasmannia sp.1	G	0.556	0.204	0.185	0.407	0.13	0.167	0.5	0.111	0.074
Wasmannia sp.2	G	0.602	0.241	0.194	0.417	0.111	0.435	0.148	0.12	0.074
