# Supplementary material for: Ecosystem-Wide Morphological Structure of Leaf-Litter Ant Communities along a Tropical Latitudinal Gradient
Source: PLoS One. 2014 Mar 26;9(3):e93049. doi: 10.1371/journal.pone.0093049 (PMC3966852; doi:10.1371/journal.pone.0093049)
Supplement: Table S3 — (A) Generalized Additive Model (GAM) results for the leaf-litter ant taxonomic and functional diversity in 26 Atlantic Forest sites. The predictors are smoothers for Latitude and Altitude. e.d.f., Estimated degrees of freedom. (B) Generalized Linear Model (GLM) results for the leaf-litter ant diversity (taxonomic) in low land Atlantic Forest sites (<400 m). The predictors are Latitude and Habitat Area. d.f., Degrees of freedom. (PDF) [file pone.0093049.s008.pdf]

Table S3-A. Generalized Additive Model (GAM) results for the leaf-litter ant taxonomic and functional diversity in 26 Atlantic Forest sites. The predictors are smoothers for Latitude and Altitude. e.d.f., Estimated degrees of freedom.

Model	Coefficients (Std. Error)		t (F)-value	P-value	Adj. R-square	e.d.f.
	Intercept	93.15 (4.22)	22.04	< 0.001	0.258	
Sobs ~ *f(Latitude)* + *f(Altitude)*	*f(Latitude)*	-	8.333	**0.008**		1
	f(Altitude)		1.388	0.250		1
Srar ~ *f(Latitude)* + *f(Altitude)*	Intercept	69.500 (2.84)	27.98	< 0.001	0.248	
	f(Latitude)		8.960	**0.006**		1
	f(Altitude)		0.697	0.412		1
Ngen ~ *f(Latitude)* + *f(Altitude)*	Intercept	32.57 (0.84)	38.6	< 0.001	0.06	
	f(Latitude)		1.035	0.320		1
	f(Altitude)		4.021	0.057		0.99
Occurrence ~ *f(Latitude)* + *f(Altitude)*	Intercept	697.77 (43.41)	16.07	< 0.001	0.491	
	f(Latitude)		6.468	0.001		3.748
	f(Altitude)		0.064	0.802		1

The dependent variable is the observed species richness (Sobs), rarefied species richness (Srar), number of genera (Ngen), and species occurrences in 50 leaf-litter $1m^2$ samples collected at each site.

Table S3-B. Generalized Linear Model (GLM) results for the leaf-litter ant diversity (taxonomic) in low land Atlantic Forest sites (< 400 m). The predictors are Latitude and Habitat Area. d.f., Degrees of freedom.

Model	Coefficients (Std. Error)		Z (t)-value	P-value	Deviance Explained (%)	d.f.
	Intercept	4.210 (0.124)	33.871	< 0.001	34.06	
Sobs ~ Latitude (*)	Slope	0.017	0.006	**0.012**		16
Srar ~ Latitude	Intercept	4.028 (0.083)	46.171	< 0.001	28.54	16
	Slope	0.011 (0.004)	2.871	**0.004**		
Ngen ~ Latitude (*)	Intercept	3.367 (0.065)	51.811	< 0.001	20.98	16
	Slope	0.006 (0.003)	2.043	0.057		
Occurrence ~ Latitude (*)	Intercept	6.384 (0.206)	30.933	< 0.001	4.38	16
	Slope	0.009 (0.010)	0.892	0.386		
Sobs ~ Latitude + Area (*)	Intercept	4.285 (0.196)	21.811	< 0.001	35.19	15
	Latitude	0.020 (0.008)	2.295	**0.036**		
	Log10 (Area)	- 0.035 (0.071)	-0.503	0.622		

The dependent variable is the observed species richness (Sobs), rarefied species richness (Srar), number of genera (Ngen), and species occurrences in 50 leaf-litter 1m^2 samples collected at each site. (*) indicates model corrected for overdispersion.
