# Supplementary material for: Ecosystem-Wide Morphological Structure of Leaf-Litter Ant Communities along a Tropical Latitudinal Gradient
Source: PLoS One. 2014 Mar 26;9(3):e93049. doi: 10.1371/journal.pone.0093049 (PMC3966852; doi:10.1371/journal.pone.0093049)
Supplement: Table S4 — (A) Multiple Predictor Models of taxonomic and functional diversities (Model AIC values) for the leaf-litter ant fauna in low (<400 m) and high (>700 m) land Atlantic forest areas. Predictors are Temperature Annual Range, Altitude (m), AET, Precipitation and Habitat Area. Values in bold indicates significant variable contributing to the model (** 0.01, *0.05). (B) Multiple Predictor Models of taxonomic and functional diversities (Model AIC values) for the leaf-litter ant fauna in low land Atlantic forest areas (<400 m). Predictors are Temperature Annual Range, Altitude (m), AET, Precipitation and Habitat Area. Values in bold indicates significant variable contributing to the model (*** 0.001, ** 0.01, *0.05, #0.1). (PDF) [file pone.0093049.s009.pdf]

**Table S4-A.** Multiple Predictor Models of taxonomic and functional diversities (Model AIC values) for the leaf-litter ant fauna in low (< 400 m) and high (>700 m) land Atlantic forest areas. Predictors are Temperature Annual Range, Altitude (m), AET, Precipitation and Habitat Area. Values in bold indicates significant variable contributing to the model (** 0.01, *0.05).

| | **Sobs** | **Srar** | **Species Occurrence** | **$FD_{PG}$** | **FRic** | **FEve** | **FDiv** | **FSpe** |
|---|---|---|---|---|---|---|---|---|
| *All Variables in the Model (AIC)* | 227.21 | 195.14 | 359.14 | 78.63 | -14.22 | -154.56 | -179.07 | -105.71 |
| *Best Model (AIC)* | 221.77 (Temp) | 193.2 (Temp + Alt + Prec) | 351.43 (Intercept) | 75.65 (AET + Prec) | -14.22 (Temp + Alt + AET + Prec + Area) | -159.53 (Temp) | -186.46 (AET) | -106.66 (Temp + Alt + AET + Prec) |
| *Effect of Variable Removed From Full Model* | | | | | | | | |
| *Temperature* | 226.43 | 194.22 | 358.74 | 76.912 | -12.334 | -155.91 | -180.49 | **-99.701*** |
| *Altitude (m)* | 225.72 | 194.49 | 357.76 | 76.741 | -12.677 | -155.90 | -180.76 | -106.333 |
| *AET* | 225.55 | 195.18 | 357.93 | 80.658 | -11.820 | -155.63 | -180.27 | **-98.697**** |
| *Precipitation* | 227.02 | **197.36*** | 357.55 | **83.561*** | -12.786 | -155.16 | -181.04 | **-101.087*** |
| *log10 (Area)* | 225.91 | 193.36 | 357.21 | 78.973 | -13.768 | -156.17 | -180.71 | -106.657 |

**Table S4-B.** Multiple Predictor Models of taxonomic and functional diversities (Model AIC values) for the leaf-litter ant fauna in low land Atlantic forest areas (< 400 m). Predictors are Temperature Annual Range, Altitude (m), AET, Precipitation and Habitat Area. Values in bold indicates significant variable contributing to the model (*** 0.001, ** 0.01, *0.05, #0.1).

| | **Sobs** | **Srar** | **Species Occurrence** | **$FD_{PG}$** | **FRic** | **FEve** | **FDiv** | **FSpe** |
|---|---|---|---|---|---|---|---|---|
| *All Variables in the Model (AIC)* | 154.38 | 133.01 | 249.53 | 49.081 | -22.326 | -106.88 | -121.74 | -69.784 |
| *Best Model (AIC)* | 150.49 (Temp + Prec) | 130.08 (Temp + Prec) | 246.34 (Intercept) | 46.58 (Temp + Prec) | -27.17 (Intercept) | -111.71 (Temp) | -127.41 (AET) | -71.63 (Temp + AET + Prec) |
| *Effect of Variable Removed From Full Model* | | | | | | | | |
| *Temperature* | **158.39*** | 133.49 | **252.83*** | 48.463 | -23.847 | -108.21 | -123.5 | **-65.116*** |
| *AET* | 152.44 | 132.04 | **250.32#** | 48.142 | -22.199 | -107.94 | -122.25 | **-65.633*** |
| *Precipitation* | 153.29 | **134.31#** | 249.09 | **51.853#** | -21.461 | -107.98 | -123.72 | -68.705 |
| *log10 (Area)* | 152.47 | 131.07 | **251.18#** | 47.107 | -23.686 | -108.35 | -123.44 | -71.633 |
