# Supplementary material for: Ecosystem-Wide Morphological Structure of Leaf-Litter Ant Communities along a Tropical Latitudinal Gradient
Source: PLoS One. 2014 Mar 26;9(3):e93049. doi: 10.1371/journal.pone.0093049 (PMC3966852; doi:10.1371/journal.pone.0093049)
Supplement: Table S5 — Partial Mantel correlations between community or functional similarity and environmental distance controlling for geographic distance (and vice versa). (PDF) [file pone.0093049.s010.pdf]

**Table S5.** Partial Mantel correlations between community or functional similarity and environmental distance controlling for geographic distance (and vice versa). Statistical significance for each partial Mantel correlation value is given in parentheses.

| | **Environmental distance** | **Geographic distance** |
|---|---|---|
| Community composition (Sorensen) | **0.573 (0.001)** | **0.579 (0.001)** |
| Functional beta diversity (MPD) | -0.212 (0.986) | 0.227 (0.036) |
| Functional beta diversity (MNTD) | **0.513 (0.001)** | **0.385 (0.01)** |
| Functional beta diversity (Phylo Sorensen) | **0.632 (P=0.001)** | **0.363 (P=0.01)** |
