# Supplementary material for: Ecosystem-Wide Morphological Structure of Leaf-Litter Ant Communities along a Tropical Latitudinal Gradient
Source: PLoS One. 2014 Mar 26;9(3):e93049. doi: 10.1371/journal.pone.0093049 (PMC3966852; doi:10.1371/journal.pone.0093049)
Supplement: Table S6 — Summary of model selection on constrained ordination models (dbRDA) that describe the influence of environmental variables on the leaf-litter ant fauna along Atlantic Forest sites. (PDF) [file pone.0093049.s011.pdf]

Table S6. Summary of model selection on constrained ordination models (dbRDA) that describe the influence of environmental variables on the leaf-litter ant fauna along Atlantic Forest sites.

	**Model**	**F**	**p-value**	**R^2**	**R^2 adj**
Community composition (Sorensen)	~ Temperature annual range + PET + Altitude + Area	4.517	**0.005**	0.462	0.360
Functional beta diversity (MPD)	~ Temperature annual range	1.336	**0.005**	0.053	0.013
Functional beta diversity (MNTD)	~ Temperature annual range + PET + Altitude	5.503	**0.005**	0.438	0.362
Functional beta diversity (Phylo Sorensen)	~ Temperature annual range + Altitude + PET + Area	4.346	**0.005**	0.456	0.352

MPD: mean pairwise distance separating taxa in two communities. Distances separating species were weighted by species abundances; MNTD: inter-community mean nearest-neighbor distance.
