# Supplementary material for: Ecosystem-Wide Morphological Structure of Leaf-Litter Ant Communities along a Tropical Latitudinal Gradient
Source: PLoS One. 2014 Mar 26;9(3):e93049. doi: 10.1371/journal.pone.0093049 (PMC3966852; doi:10.1371/journal.pone.0093049)
Supplement: Table S7 — Summary of GAMs to examine the relationship between the morphological structure of the leaf-litter ant assemblages and latitude in the Atlantic Forest. (PDF) [file pone.0093049.s012.pdf]

Table S7. Summary of GAMs to examine the relationship between the morphological structure of the leaf-litter ant assemblages and latitude in the Atlantic Forest.

**Model**	**Estimated df**	**Reference df**	**F**	**p-value**	**Adj. R-square**	**Deviance explained (%)**
			**Unconstrained Species Pool**			
MPD ~ Lat						
	1.514	1.872	5.709	0.011	0.256	30.1
MNTD ~ Lat	1.856	2.333	10.83	0.001	0.498	53.6
			**Constrained Species Pools**			
MPD ~ Lat	1	1	1.237	0.277	0.009	4.9
MNTD ~ Lat	1.114	1.219	2.065	0.162	0.051	9.27
