# Supplementary material for: Ecosystem-Wide Morphological Structure of Leaf-Litter Ant Communities along a Tropical Latitudinal Gradient
Source: PLoS One. 2014 Mar 26;9(3):e93049. doi: 10.1371/journal.pone.0093049 (PMC3966852; doi:10.1371/journal.pone.0093049)
Supplement: Table S8 — Summary of GAMs to examine the relationship between the morphological structure of the leaf-litter ant guilds and latitude in the Atlantic Forest. (PDF) [file pone.0093049.s013.pdf]

Table S8. Summary of GAMs to examine the relationship between the morphological structure of the leaf-litter ant guilds and latitude in the Atlantic Forest. Significant (P < 0.05) and marginally significant (P < 0.1).

**Model**	**Estimated df**	**Reference df**	**F**	**p-value**	**Adj. R-square**	**Deviance explained (%)**
**MPD, Unconstrained Species Pool**						
Fungus-growers	1	1	8.277	0.008	0.225	25.6
Generalists	1.776	2.23	5.039	0.013	0.292	34.2
Medium-size hypogaeic predators	2.038	2.543	2.616	0.090	0.208	28.8
**MNTD, Unconstrained Species Pool**						
Fungus-growers	3.137	3.881	2.71	0.058	0.256	34.9
Medium-size epigaeic predators	1.341	1.61	9.649	0.001	0.342	37.7
Specialized predators	2.541	3.159	3.764	0.026	0.344	42
**MPD, Constrained Species Pool**						
Medium-size epigaeic predators	1	1	4.488	0.044	0.122	15.8
Medium-size hypogaeic predators	1.957	2.445	2.48	0.103	0.193	27.2
**MNTD, Constrained Species Pool**						
Fungus-growers	2.788	3.467	2.32	0.095	0.216	30.3
Small-size hypogaeic generalists	3.999	4.899	7.393	0.001	0.582	64.9
Small-size hypogaeic predators	1.412	1.72	9.817	0.001	0.362	39.8
